# A Versatile Human Intestinal Organoid-Derived Epithelial Monolayer Model for the Study of Enteric Pathogens

**DOI:** 10.1128/spectrum.00003-21

**Published:** 2021-06-09

**Authors:** Kourtney P. Nickerson, Alejandro Llanos-Chea, Laura Ingano, Gloria Serena, Alba Miranda-Ribera, Meryl Perlman, Rosiane Lima, Marcelo B. Sztein, Alessio Fasano, Stefania Senger, Christina S. Faherty

**Affiliations:** a Mucosal Immunology and Biology Research Center, Division of Pediatric Gastroenterology and Nutrition, Massachusetts General Hospital, Boston, Massachusetts, USA; b Department of Pediatrics, Harvard Medical School, Boston, Massachusetts, USA; c Center for Vaccine Development and Global Health, Department of Pediatrics, University of Maryland School of Medicine, Baltimore, Maryland, USA; Broad Institute

**Keywords:** *Shigella*, *Salmonella*, *Escherichia coli*, infection models, human, intestinal, organoid, enteroid, HIODEM, DAPT, RANKL, epithelial monolayer, enterocytes, goblet cells, mucus, M cells

## Abstract

Gastrointestinal infections cause significant morbidity and mortality worldwide. The complexity of human biology and limited insights into host-specific infection mechanisms are key barriers to current therapeutic development. Here, we demonstrate that two-dimensional epithelial monolayers derived from human intestinal organoids, combined with *in vivo-*like bacterial culturing conditions, provide significant advancements for the study of enteropathogens. Monolayers from the terminal ileum, cecum, and ascending colon recapitulated the composition of the gastrointestinal epithelium, in which several techniques were used to detect the presence of enterocytes, mucus-producing goblet cells, and other cell types following differentiation. Importantly, the addition of receptor activator of nuclear factor kappa-B ligand (RANKL) increased the presence of M cells, critical antigen-sampling cells often exploited by enteric pathogens. For infections, bacteria were grown under *in vivo-*like conditions known to induce virulence. Overall, interesting patterns of tissue tropism and clinical manifestations were observed. Shigella flexneri adhered efficiently to the cecum and colon; however, invasion in the colon was best following RANKL treatment. Both Salmonella enterica serovars Typhi and Typhimurium displayed different infection patterns, with *S*. Typhimurium causing more destruction of the terminal ileum and *S*. Typhi infecting the cecum more efficiently than the ileum, particularly with regard to adherence. Finally, various pathovars of Escherichia coli validated the model by confirming only adherence was observed with these strains. This work demonstrates that the combination of human-derived tissue with targeted bacterial growth conditions enables powerful analyses of human-specific infections that could lead to important insights into pathogenesis and accelerate future vaccine development.

**IMPORTANCE** While traditional laboratory techniques and animal models have provided valuable knowledge in discerning virulence mechanisms of enteric pathogens, the complexity of the human gastrointestinal tract has hindered our understanding of physiologically relevant, human-specific interactions; and thus, has significantly delayed successful vaccine development. The human intestinal organoid-derived epithelial monolayer (HIODEM) model closely recapitulates the diverse cell populations of the intestine, allowing for the study of human-specific infections. Differentiation conditions permit the expansion of various cell populations, including M cells that are vital to immune recognition and the establishment of infection by some bacteria. We provide details of reproducible culture methods and infection conditions for the analyses of *Shigella*, *Salmonella*, and pathogenic Escherichia coli in which tissue tropism and pathogen-specific infection patterns were detected. This system will be vital for future studies that explore infection conditions, health status, or epigenetic differences and will serve as a novel screening platform for therapeutic development.

## INTRODUCTION

Over 10 million pediatric deaths occur annually, with over half of these deaths resulting from microbial infection (1). Despite improvements in hygiene, availability of clean water, and access to treatment, few preventive therapies exist and pediatric mortality rates remain high (1–5). Gastrointestinal (GI) pathogens such as *Shigella*, Salmonella, and Escherichia coli cause diarrhea, dysentery, and even sepsis in some instances (6–10). Many of these pathogens are human restricted, rendering traditional laboratory models insufficient for understanding disease pathologies in humans. In fact, the complexities of human biology coupled with human-specific infection patterns are key barriers to understanding pathogenic mechanisms for successful vaccine development.

To overcome these barriers, we further developed methodology to transition human intestinal stem cells from spheroid cultures into single two-dimensional (2D) cell monolayers that recapitulate the various cell types of the GI tract (11) and answer critical questions regarding how human-specific pathogens interact with host cells. Here, we present detailed protocols to prepare human intestinal organoid-derived epithelial monolayers (HIODEM) isolated from the ileum, cecum, and ascending colon to test infection with *Shigella*, Salmonella, and pathogenic E. coli. Monolayers were composed of enterocytes, goblet cells, and other cell types of the various intestinal segments. Through ligand stimulation, the monolayer system was modified to promote expansion of the microfold (M) cell population of the follicle-associated epithelium often exploited by bacterial pathogens to gain access to the epithelium (12, 13). Both adherence and invasion of the three GI segment-derived models were examined with the various pathogens, and the role of M cells in enteric infections was evaluated for *Shigella* and Salmonella. We also utilized bacterial growth conditions that replicate the host environment to enhance the virulence of the pathogens prior to infection. In all, this combined methodology has significant potential to transition current research methods into the most human-specific, *in vivo-*like model to study bacterial pathogenesis and preclinically evaluate therapeutic candidates, potentially leading to paradigm-shifting approaches to vaccine or therapeutic development.

## RESULTS

### The HIODEM model for studying enteric pathogenesis.

Organoids were derived from tissue biopsy specimens collected from the terminal ileum, cecum, and ascending colon and subcultured for monolayer generation. After isolation and propagation, crypt stem cells were dissociated into single cells and seeded onto transwell inserts. Monolayers reached confluence in 7 to 10 days, during which transepithelial electrical resistance (TEER) was monitored to indicate formation of functional barriers (14). TEER readings were highest in the terminal ileum, averaging 1,010 ± 112 Ω·cm^2^ with 0.33-cm^2^ transwells, whereas cecum monolayers averaged 490 ± 31 Ω·cm^2^ and the colon had much lower TEER values, averaging 209 ± 2.7 Ω·cm^2^ (Table 1). Following the stabilization of TEER readings, monolayers were treated with the γ-secretase inhibitor N-[N-(3,5-difluorophenacetyl)-l-alanyl]-S-phenylglycine t-butyl ester (DAPT) (11, 15), with or without the receptor activator of nuclear factor kappa-Β ligand (RANKL) (16) at physiological (100 ng/ml) or supraphysiological (500 ng/ml) doses for 24 or 48 h to ensure differentiation and provide the immune stimulation to induce M cell expression (16–18).

**TABLE 1:** Average TEER values from this study and reported in the literature^*a*^

Cell type/tissue	Origin	Support	TEER value (Ω·cm^2^ ± SEM)	Reference or source
Biopsy ileum	Terminal ileum	TW	1,010 ± 112	This study
Biopsy cecum	Cecum	TW	490 ± 31	This study
Biopsy colon	Ascending colon	TW	209 ± 2.7	This study
Caco-2*	Colorectal adenocarcinoma	TW	400	104
Caco-2		TW	400	105
Caco-2		TW	600	106
Caco-2		TW	1,000	107
Caco-2		TW	900 ± 23	108
Caco-2/HT-29*	Coculture model	TW	300–400	109
IPEC-J2*	Porcine jejunum	TW	1,500	110
IPEC-J2		TW	1,000–5,000	111
hiPS-ELC	Human embryonic pluripotent stem cells	TW	250	105
hiPS-ELC		TW	100	112
hiPS-ELC		TW	900	113
hInEpCs	Small intestine	TW	1,500	113
hInEpCs		TW	400	114
FEnS/AEnS	Fetal intestines and adult duodenum	TW	400–600	15
AEnS	Duodenum	TW	600–1,000	71
hIECs/colonic fibroblasts	Colon	TW	250	115
hiPS-ileal ELC	Ileal	TW	400	11
hiPS-rectal ELC	Rectal	TW	395	11
hIECS	Small intestine	TW	98.9 ± 17.5	108

a Abbreviations: IPEC, intestinal porcine enterocyte cells; hiPS, human induced pluripotent stem cells; ELC, enterocyte-like cells; hInEpCs, human primary intestinal epithelial cells (commercially available); FEnS, fetal-derived enterospheres; AEnS, adult-derived enterospheres; hIEC, human intestinal epithelial cell; TW, transwell. *, cell lines.

**FIG 1:**
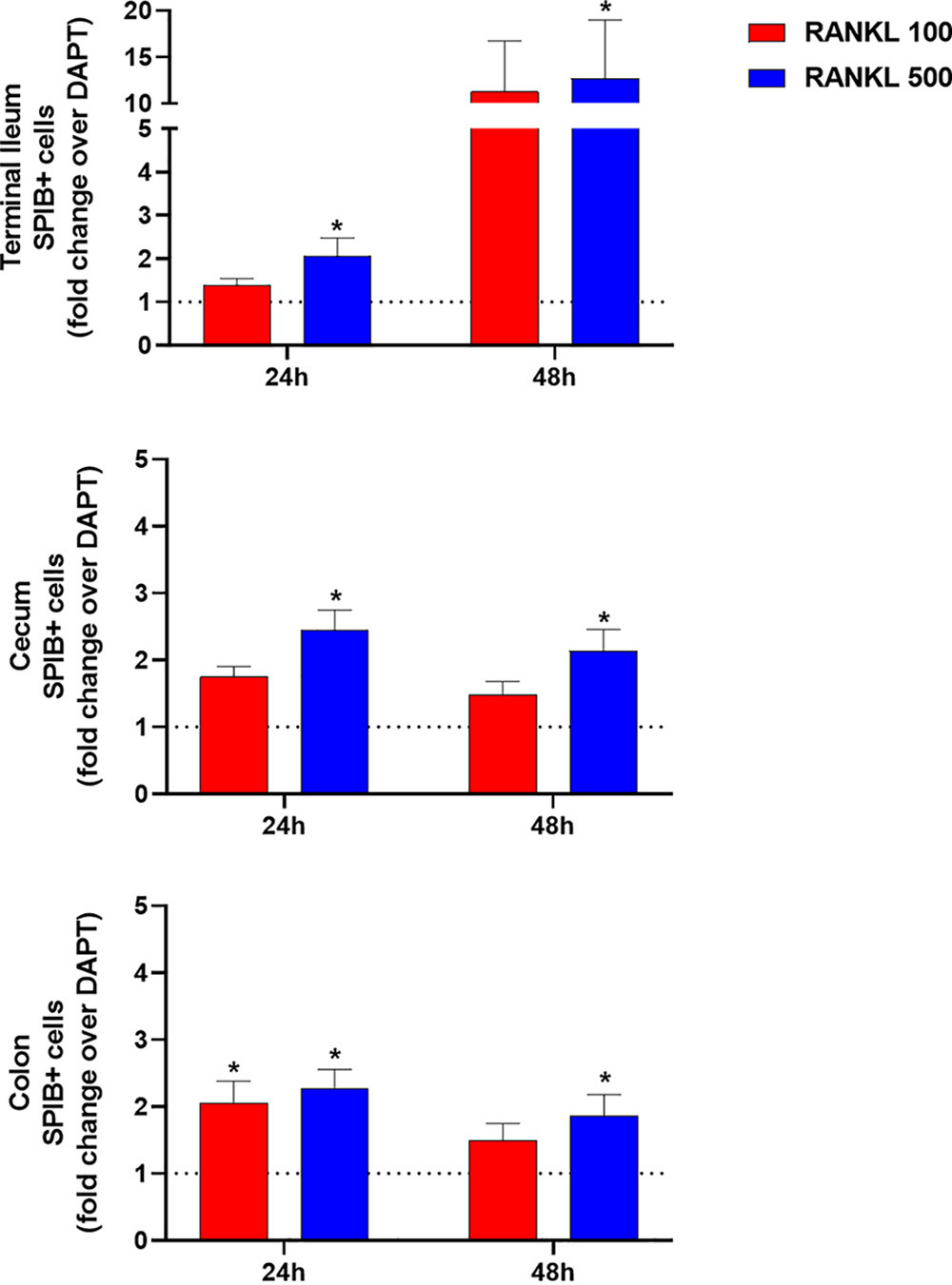
Flow cytometry analyses identify an increase in SPIB^+^ expression following RANKL treatment. The increase in SPIB^+^ expression, which is indicative of M cell expression, after 24 or 48 h of RANKL treatment is represented as fold change of SPIB^+^ cells in tissues treated with RANKL (either DAPT + RANKL, 100 ng/ml, red bars, or DAPT + RANKL, 500 ng/ml, blue bars) over the matching tissues treated only with DAPT (represented by the dotted line) for each tissue. Statistical significance was determined by paired Friedman test for the DAPT + RANKL (100 or 500 ng/ml) differentiation compared to DAPT only differentiation of the matched originating tissue (*, *P* < 0.05). A total of 4 biological replicate experiments were analyzed for the terminal ileum and cecum, and 6 biological replicates were analyzed for the ascending colon. For each biological sample, an entire 12-well plate was trypsinized, and the cells were pooled into one sample tube for staining and analysis.

**FIG 2:**
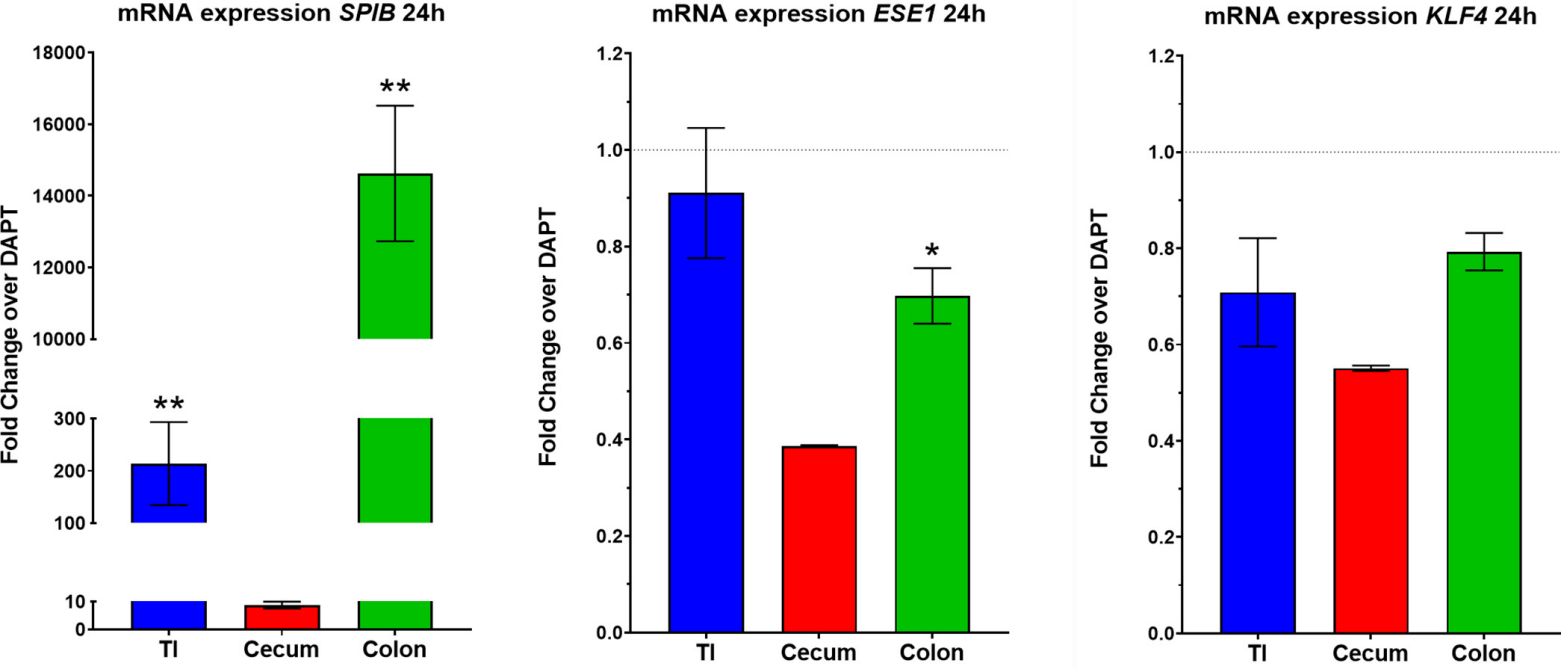
RT-qPCR analyses demonstrate significantly induced *SPIB* gene expression following RANKL treatment. Gene expression for *SPIB*, *ESE1*, and *KLF4* was measured following 24 h of DAPT + 100 ng/ml RANKL or DAPT only treatment for the terminal ileum (TI, blue, *n* = 6), cecum (red, *n* = 3), and colon (green, *n* = 6). Data are expressed as DAPT + RANKL fold change over the DAPT only treatment, shown as the average fold change ± standard errors of the means. All data were normalized to expression from the *18S* housekeeping gene. Statistical significance was determined with the Mann-Whitney unpaired *t* test of the Δ*C_T_*. Data were considered significant at a *P* value of <0.05 (*, <0.05; **, <0.01). Please note the different *y* axes for each graph.

**FIG 3:**
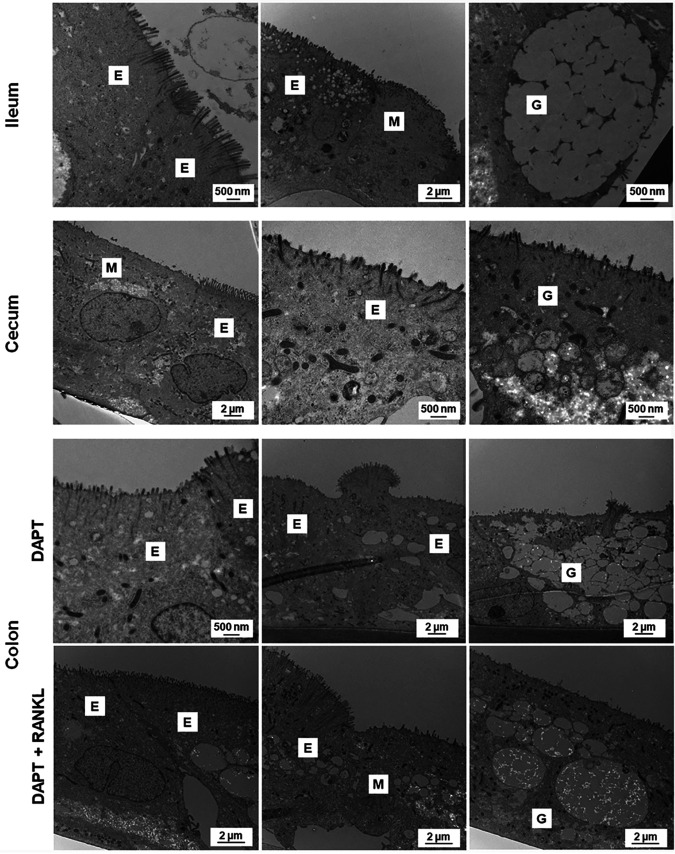
Transmission electron microscopy analysis of the HIODEM models. Mature monolayers derived from the terminal ileum (top), cecum (middle), or colon (bottom) were evaluated by TEM to visualize the cell types present, monolayer quality, and barrier formation. All models displayed characteristic features of enterocytes (E), goblet cells (G), and M cells (M). For the colon, differentiation treatments with DAPT and DAPT + RANKL were compared to visualize the presence of M cells. Magnifications range from 5,000× to 25,000×, and the corresponding scale bars are provided.

Several methodologies were used to characterize the differentiated monolayers, including flow cytometry, reverse transcription quantitative PCR (RT-qPCR), and transmission electron microscopy (TEM). We focused on ensuring the presence of enterocytes, mucus-producing goblet cells, and M cells, with other cell markers evaluated by RT-qPCR only. First, for flow cytometry, mature monolayers were examined using antibodies against the transcription factors ESE1 for enterocytes (19), KLF4 for goblet cells (20), and SPIB for M cells (17). The analyses confirmed the presence of the respective cellular markers and enabled us to estimate the cellular populations for each cell type (see Fig. S1 in the supplemental material). Specifically, for the M cell phenotype, RANKL treatment increased the percentage of SPIB^+^ cells relative to the DAPT only treatment across all tissue types with both doses of RANKL for 24 and 48 h (Fig. 1). Second, to confirm the induction of the *SPIB* gene with RANKL treatment, we analyzed gene expression at 24 h following 100 ng/ml RANKL treatment (Fig. 2). The RT-qPCR analyses revealed significant induction of *SPIB* expression with the DAPT plus RANKL treatment relative to the DAPT only treatment, with no or minor changes in expression for *ESE1*, *KLF4*, and other cellular markers (Fig. 2 and Table 2). It is important to note that expression of *ESE1*, *KLF4*, and the other genes was detected under all conditions (data not shown), but the changes in expression were not significantly altered in DAPT plus RANKL treatment compared to DAPT only treatment. Finally, mature monolayers were evaluated by TEM to visualize and confirm the various cell types, monolayer quality, and barrier formation (Fig. 3). Characteristic features of enterocytes, goblet cells, and M cells were visualized, which included the presence of microvilli for enterocytes, secretory granules for goblet cells, and absent or disorganized microvilli with displaced nuclei for M cells (21–23). In all, the data demonstrated that multiple cellular populations were present in the HIODEM models evaluated under the differentiation conditions described above and that RANKL treatment increased the presence of M cells.

**TABLE 2:** Gene expression analyzed by RT-qPCR in models treated with 100 ng/ml RANK + DAPT for 24 h

Gene^*a*^	Terminal ileum		Cecum		Colon	
	FC^*b*^	*P* value^*c*^	FC	*P* value	FC	*P* value
*LRG5*	1.1	NS	4.7	NS	0.4	<0.005
*SPIB*	213.9	<0.005	8.7	NS	14,625.1	<0.005
*KLF4*	0.7	NS	0.6	NS	0.8	NS
*ESE1*	0.9	NS	0.4	NS	0.7	<0.05
*MUC2*	2.6	NS	0.2	NS	1.2	NS
*CGA*	0.3	NS	0.5	NS	1.7	NS
*NEUROD*	BDL		BDL		1.2	NS
*SPDEF*	0.8	NS	0.4	NS	0.3	<0.005
*SOX9*	1.2	NS	0.3	NS	1.3	<0.05
*LYZ*	1.1	NS	0.2	NS	0.6	<0.005
*OCLDN*	1.2	NS	0.4	NS	1.1	NS
*SIM*	1.1	NS	0.2	NS	1.0	NS

a Please refer to Materials and Methods for information on each gene.

b Fold change (FC) reflects the fold change in gene expression in the 100 ng/ml RANKL + DAPT treatments relative to the DAPT treatment at 24 h. BDL, below detection level. Note that the *SPIB* expression levels correspond to the data presented in Fig. 2.

c Statistical significance determined with the nonparametric Mann-Whitney test. NS, not significant.

### Robust *Shigella* infection requires colonic M cells.

**FIG 4:**
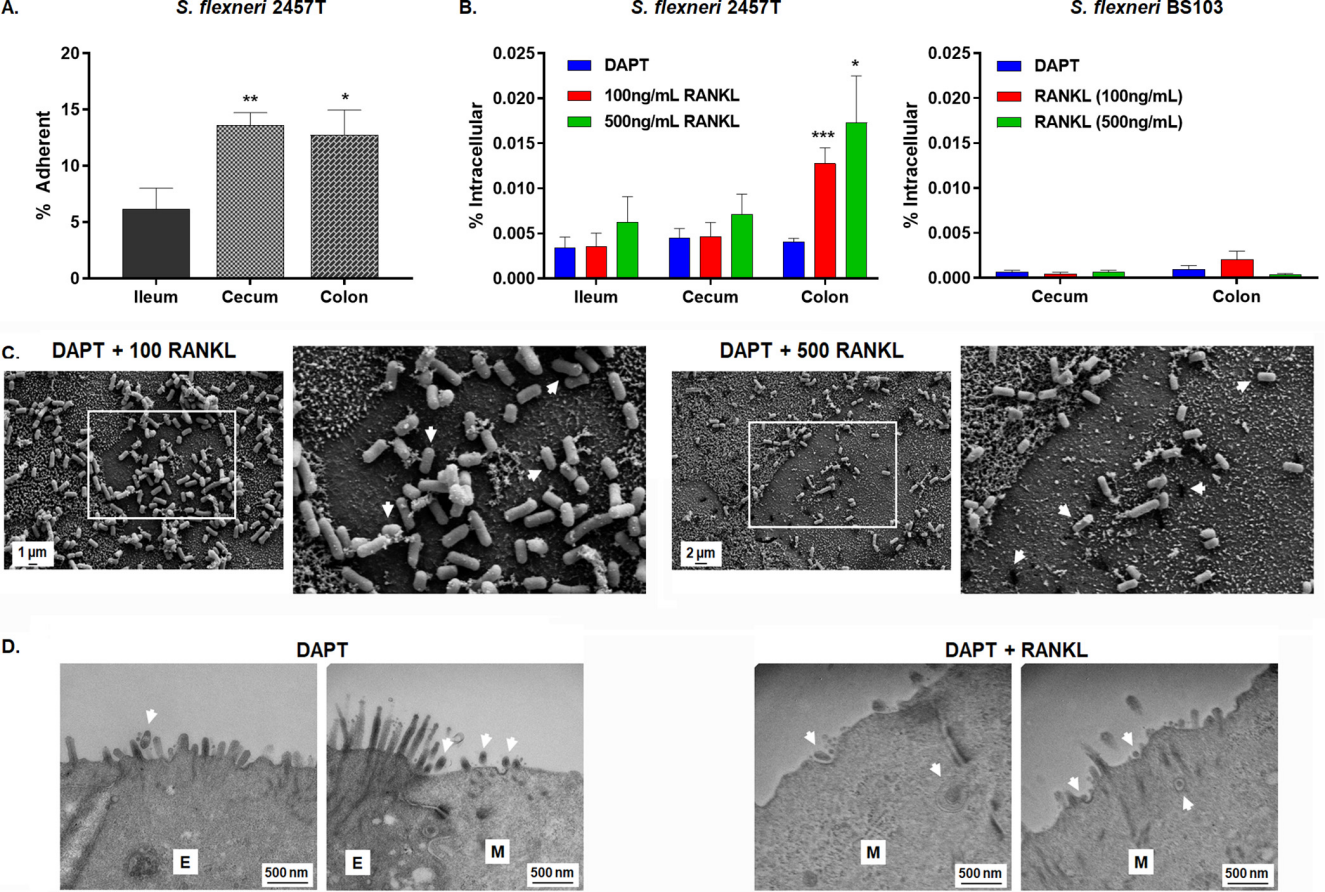
*Shigella* infection analysis reveals a tissue tropism for the colon. Infection analyses were performed with at least three biological replicates, in which three technical replicates were present for each experiment. Imaging analysis was performed on biological or technical independent samples relative to the plotted infection data. For plotted data, please note the different *y* axis scales. Statistical significance was determined by the Student's *t* test of the indicated comparisons (*, *P* < 0.05; **, *P* < 0.01; ***, *P* < 0.001). (A) Ileum-, cecum-, and colon-derived HIODEM models were infected with S. flexneri strain 2457T, and the percentage of the inoculum adherent to the monolayer surface was determined. S. flexneri was adherent to all three tissue locations with a significant increase in adherence to the cecum- and colon-derived HIODEMs relative to ileum-derived HIODEMs. Statistical analyses compared the ileum adherence rates to the cecum or colon. (B) S. flexneri invasion analysis of the HIODEM models. Colon-derived HIODEMs differentiated with DAPT and RANKL had nearly three times as many intracellular S. flexneri strain 2457T in colonic RANKL-treated HIODEMs. Statistical analyses compared the DAPT-treated monolayers relative to the two DAPT + RANKL treatments for each set of models (ileum, cecum, and colon). The noninvasive strain BS103 did not have significant recovery titers following gentamicin treatment in the cecum or colon HIODEMs despite M cell differentiation, which validated the invasion data for 2457T. (C) SEM of colon-derived HIODEMs differentiated with DAPT + RANKL at either 100 ng/ml or 500 ng/ml concentrations. Bacterial adherence to both enterocytes and M cells, with cell surfaces lacking microvilli, were observed. Magnification ranged from 5,000× to 7,000×, with 2-μm and 1-μm scale bars, respectively. Enlarged portions of the images are denoted by the white boxes, and arrows point to areas of various stages of bacterial translocation on the surface of the M cells. Please note that not all areas are highlighted. (D) Transmission electron micrographs of DAPT and DAPT + RANKL-differentiated colonic monolayers infected with S. flexneri reveal bacterial association with the apical surface of enterocytes (E) and M cells (M). Areas with bacterial cells at the cellular surface or translocating through M cells are highlighted by the arrows. Magnification for all images is 50,000× with 500-nm scale bars noted.

To validate the site of *Shigella* infection, we analyzed wild-type S. flexneri strain 2457T infection in the terminal ileum-, cecum-, and colon-derived HIODEM by assaying for adherence and invasion. Prior to infection, S. flexneri was cultured in a combination of bile salts and glucose to replicate small intestinal transit (24–26). For adherence, approximately 5% of the bacterial inoculum adhered to ileum-derived HIODEM, while the adherence rates for cecum- and colon-derived HIODEM models were nearly triple (∼12 to 15%; Fig. 4A). For invasion, rates were consistent across the models under most treatments; however, colon-derived monolayers differentiated with RANKL to promote the maturation of M cells consistently had a 3-fold increase in invasion (Fig. 4B). A virulence plasmid-cured, noninvasive strain of S. flexneri (strain BS103 [27]) was used as a negative control for invasion and was unable to invade the models under any conditions. Scanning electron microscopy (SEM) of S. flexneri-infected colonic models demonstrated attachment to the apical surface of the cells (Fig. 4C). The S. flexneri adherence pattern occurred throughout the surface of the monolayer. Additionally, shadowed areas beneath adherent bacteria appeared on cells lacking microvilli in the RANKL-treated samples, accompanied by perforations in other parts of the cells. These observations appear to be visual representations of M cell transit required for *Shigella* invasion (28). Cross-section TEM verified these observations with visualization of invading bacteria localized to M cells (Fig. 4D). Finally, to further validate the model, S. flexneri*-*infected colon HIODEM monolayers were evaluated for interleukin-8 (IL-8) and lactate dehydrogenase (LDH) release (Fig. S2), since *Shigella* infection is accompanied by IL-8 secretion (29, 30) and inhibition of epithelial cell death (31, 32). Significant IL-8 secretion from S. flexneri infection was observed, while mock-treated cells had no detectable levels of IL-8. Additionally, very low levels of LDH were detected for S. flexneri-infected monolayers with no significant difference from mock infection.

### Serovar-specific aspects of Salmonella infection are revealed using the HIODEM model.

**FIG 5:**
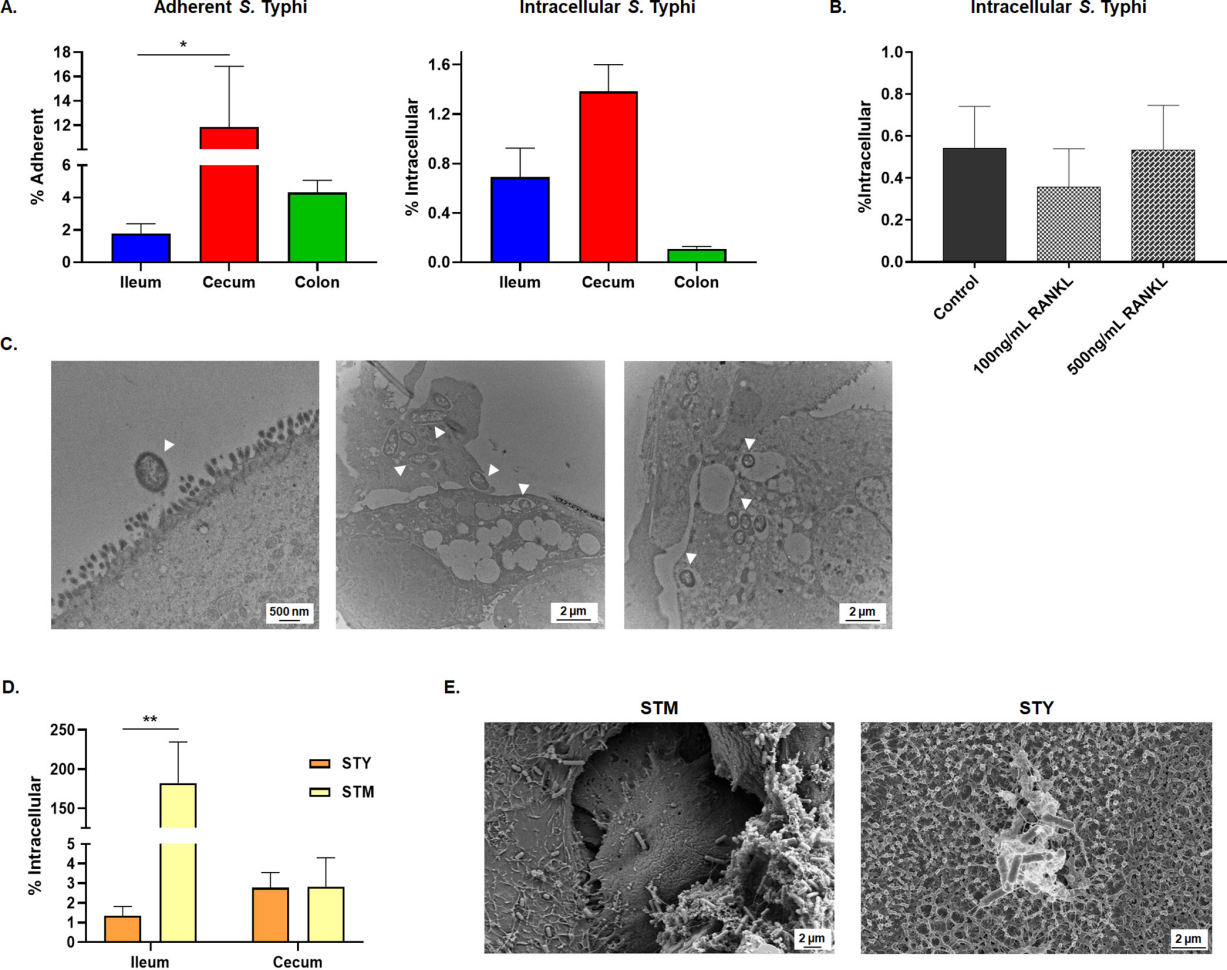
HIODEM model demonstrates Salmonella serovar-specific infection patterns. Infection analyses were performed with at least three biological replicates, in which three technical replicates were present for each experiment. Imaging analysis was performed on biological or technical independent samples relative to the plotted infection data. For plotted data, please note the different *y* axis scales. Statistical significance was determined by the Student's *t* test of the indicated comparisons (*, *P* < 0.05; **, *P* < 0.01). (A) Salmonella *enteric* serovar Typhi strain Ty2 adhered to ileum-, cecum-, and colon-derived HIODEMs, with the greatest rate detected in the cecum monolayers. Invasion was prevalent in the ileum- and cecum-derived HIODEMs, with a modest increase in the cecum monolayers. (B) RANKL treatment of the ileum HIODEMs did not result in significant differences in the rate of invasion. (C) TEM analysis of *S*. Typhi invasion in the ileum HIODEMs. Magnification ranged from 10,000× to 25,000×, with 2-μm and 500-nm scale bars, respectively. Arrows point to bacteria; note that not all bacterial are highlighted. (D) Salmonella enterica serovar Typhimurium strain SL1344 (STM) preferentially invades ileum HIODEMs at significantly higher rates relative to *S*. Typhi (STY). (E) Scanning electron micrographs of STM- or STY-infected ileum HIODEMs. STM infection is accompanied by cell rounding and barrier destruction, whereas STY infection maintains barrier function and showed bacterial association with the microvilli of enterocytes. Magnifications range from 7,000× to 14,000×, with 2-μm scale bars highlighted for each image. Refer to Fig. S3 for additional images.

To demonstrate versatility of the HIODEM model, monolayers derived from ileum, cecum, and colon tissue were used to examine adherence and invasion of wild-type Salmonella enterica serovar Typhi strain Ty2 and serovar Typhimurium strain SL1344. Salmonella Typhi infection was more prevalent in the cecum, with a higher rate of adherence and a modest increase in invasion relative to the ileum (Fig. 5A). Furthermore, infection of ileum HIODEMs treated with RANKL to promote M cell maturation did not result in a significant increase in intracellular bacteria (Fig. 5B). Transmission electron microscopy of infected HIODEM cells revealed *S*. Typhi associated with the enterocyte surface, remodeled the host cytoskeleton, or was contained within intracellular vesicles (Fig. 5C), while bacterial association with secreted mucus was also observed following immunostaining analysis (Fig. S3). Interestingly, comparison of *S*. Typhi to *S*. Typhimurium infections revealed serovar-specific infection patterns (Fig. 5D and E). Unlike *S*. Typhi, *S*. Typhimurium infected and replicated robustly within ileum monolayers, with significant destruction of the monolayers revealed upon SEM analysis. Like the TEM, the SEM analysis of the *S*. Typhi-infected monolayers showed bacteria interacting with enterocytes through bacterial surface structures binding to microvilli, with an overall pattern of surface association to intact monolayers. These results both verify our previous findings for *S*. Typhi infection (33) and demonstrate distinctions in serotype-specific infection patterns along different segments of the human GI tract.

### Model validation with pathogenic Escherichia coli.

**FIG 6:**
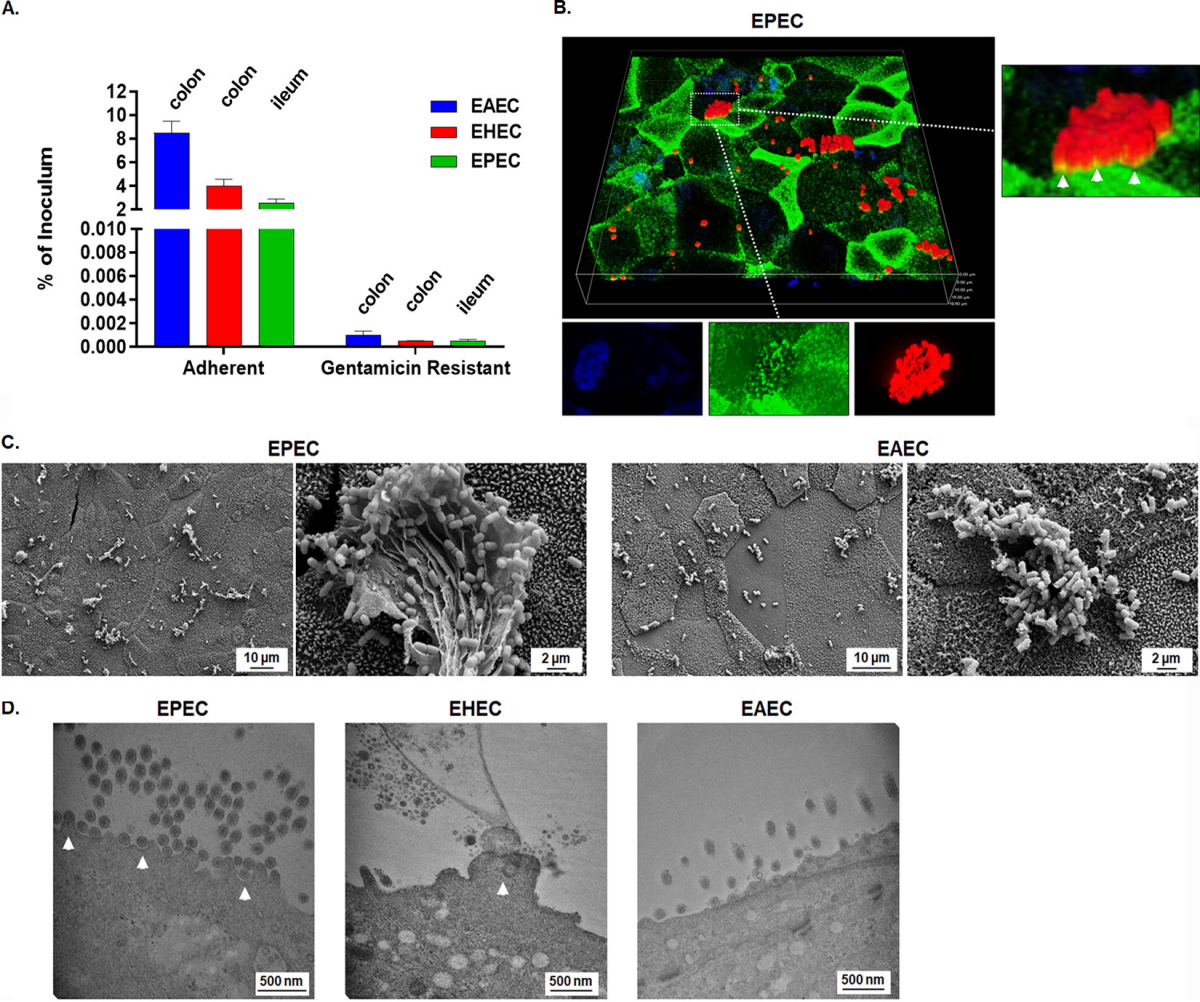
HIODEM supports infection with different Escherichia coli pathovars. (A) Enteroaggregative (EAEC strain 042), enterohemorrhagic (EHEC O157:H7 strain 933), and enteropathogenic (EPEC strain 2348/69) E. coli adhere to the surface of HIODEMs without significant invasion detected. Colon-derived HIODEMs were used for EHEC and EAEC, while ileum-derived HIODEMs were used for EPEC. (B) Three-dimensional (3D) confocal reconstructions of ileum HIODEM immunostained for actin (green), EPEC (red), and nuclei (blue) show recruitment of actin to the base of adherent bacteria in ileum-derived HIODEM. Magnification of the full image is 40×, with the height and width of the viewing plane at 141.3 μm and the depth at 19.6 μm. The image inset on the right provides a higher resolution of bacterial adherence in which arrows point to areas of bacterial and actin colocalization (yellow signal). The individual channels below the 3D image are top-down views of the same field to further highlight actin colocalization with the bacteria. (C) SEM of EPEC- and EAEC-infected monolayers demonstrate bacterial association with the host cells, aggregation of bacteria, and clusters of bacteria embedded in mucus. Magnifications range from 2,000× to 10,000×, with 10-μm and 2-μm scale bars, respectively. Ileum-derived HIODEM was used for EPEC, and colon-derived HIODEM was used for EAEC. (D) TEM images of infecting EPEC, EHEC, and EAEC. Magnifications range from 50,000× to 60,000×, with 500-nm scale bars indicated. White arrows indicate pedestal formation for EPEC and EHEC. Ileum-derived HIODEM was used for EPEC, and colon-derived HIODEMs were used for EHEC and EAEC.

Because the goal of the HIODEM system is to provide the most human-specific physiological model to analyze multiple enteric pathogens, validation of pathogenic E. coli infection would help support use of the model for a variety of bacteria. Since *Shigella* and Salmonella are both invasive pathogens, we decided to test the efficacy of our model on adherent, noninvasive pathogens while also reproducing similar analyses performed by another group (11). Enteropathogenic (EPEC strain 2348/69), enterohemorrhagic (EHEC O157:H7 strain 933), and enteroaggregative (EAEC strain 042) E. coli strains infect different locations of the intestine (i.e., ileum [34], colon [35], and colon [36], respectively). The ability of EPEC, EHEC, and EAEC to adhere or resist gentamicin was determined using organoids derived from the appropriate anatomical site specific to each pathogen in which the HIODEM monolayers were treated with DAPT only. Robust adherence for the three pathogens was detected in colon and ileum organoids; however, recoveries were minimal following gentamicin treatment (Fig. 6A). To confirm these data, confocal immunofluorescence analysis (Fig. 6B), SEM (Fig. 6C), and TEM (Fig. 6D) were performed. EPEC and EHEC adhered to the apical surface of the monolayers in tight association and indications of pedestal formation (37) were present, whereas EAEC adhered in an aggregative fashion (38). Interestingly, we found that the E. coli pathovars displayed a “hot-spot” infection pattern that was reproducible and consistent when imaged by various microscopy analyses. These results confirm the efficacy of the HIODEM model in recapitulating known phenotypes associated with pathogenic E. coli (11) and demonstrate the ability of the model to accommodate different pathogens for human cell infection analyses.

## DISCUSSION

The use of human-derived intestinal organoids to study bacterial pathogenesis has recently increased (39–42). Prior to this advancement, most enteric pathogenesis studies were limited to immortalized cell lines and/or animal models. While traditional models have provided key insights into our understanding of enteric bacterial pathogens, successful vaccine development has been ineffective or limited (43–45). Animal models do not faithfully replicate the human GI tract (46–50), while immortalized cell lines have genetic abnormalities, dysregulated cell signaling pathways, and varied differentiation statuses depending on the cell line and the culturing conditions used (22, 51–53). Thus, there is a significant need to utilize human-specific infection models to improve our understanding of pathogenesis and to facilitate the development of efficacious vaccines against *Shigella*, Salmonella, and pathogenic E. coli. Intestinal-derived organoid models provide a pluripotent platform to study host-pathogen interactions, in which differentiation reagents ensure the presence of multiple cell types of the human epithelium, while regional specificity can be retained by obtaining biopsy specimens from different segments of the GI tract. Given the complexity of the methodology and reagents, we have provided a detailed protocol of our procedure (see the supplemental material) to share with the research community and facilitate host-pathogen studies.

Organoid-derived epithelial monolayer models like the HIODEM have enabled infection analyses by providing a process by which pathogens can directly interact with the apical side of the epithelium (11, 24, 33, 54–58). While three-dimensional organoid systems are available (40, 59–63) and “apical-out” organoid systems have been developed (64), HIODEM and other 2D systems offer easily accessible monolayers for pathogenesis studies in which apical and basolateral cytokine secretion profiles and changes in TEER can be examined in a standard transwell culture setting. Similar to traditional polarized models with Caco-2 and T84 cells (65–67), the polarized monolayer of HIODEM allows for a robust infection without need of centrifuging bacteria onto the cells, which is a common practice for infection analyses performed with standard, nonpolarized cell lines (68–70). Finally, the high-throughput nature of the multiwell plate formats enables multiple monolayers to be assayed simultaneously. Possible comparative analyses include various bacterial strains or mutants, infection conditions, ligand treatment, or even screening platforms for therapeutic candidates (23). The possibilities for therapeutic evaluation are amplified by the ability to develop organoids from donors with different genetic backgrounds, environmental exposure, age, disease status, or tissue site. These factors are captured and maintained in the organoid cultures (15, 71, 72), thereby increasing the potential of the model for patient stratification and precision medicine applications.

For our HIODEM system, medium composition and inhibitors were carefully chosen to maintain the stem cell phenotype during the organoid culturing phase, initiate differentiation when seeded onto monolayers, and promote terminal differentiation in the last 48 h of monolayer culture prior to use (see the supplemental material). Removal of the A 83-01 inhibitor from the media when shifting from the organoid culture (1:1 + A 83-01 + Y-27632) to the monolayer culture (1:1 + Y-27632) promotes cell attachment to the transwells (S. Senger’s observations). As the monolayer culturing progresses, TEER measurements monitor polarization of the models, which will vary depending on the tissue of origin. As noted in Table 1, our TEER values were consistent with published values for other cell-based models on transwell systems in which measurements are highest in the proximal intestine and decrease toward the colon. Passage of fluorescein isothiocyanate (FITC) dextran from the apical medium to the basolateral medium is another method to confirm the TEER readings and integrity of the barrier, as we have previously performed (15, 33, 71). Finally, transitioning the cells from the monolayer growth medium (apical and basolateral 1:1 + Y-27632) to terminal differentiation medium (apical complete Dulbecco’s modified Eagle medium [cDMEM]/F12 plus DAPT, basolateral 1:1) prior to infection experiments promotes final differentiation, since the factors that maintain stemness are significantly reduced and only present on the basolateral side to replicate *in vivo* environments (73). Thus, the removal of apical stem cell factors in conjunction with the addition of DAPT is a critical signal that prompts the cells to differentiate and mature into a tissue-like model. It is important to note that each HIODEM model retains the cellular programming from the site of origin, i.e., ileum, cecum, or colon, despite the overall pluripotent state of the stem cells (11, 15, 71).

One of our goals was to develop a physiologically relevant infection model for *Shigella.* Given the infection paradigm in which *Shigella* requires the presence of antigen-sampling M cells to access the basolateral pole for invasion of colonic epithelial cells (28), we sought to utilize a reagent that promotes the differentiation of M cells. We used RANKL based on previous studies (74, 75). The RANKL treatment resulted in significant induction of SPIB expression as detected by flow cytometry (Fig. 1) and RT-qPCR (Fig. 2) as well as increased appearance of the M cell phenotype upon microscopic examination of the monolayers (Fig. 3), which included displaced nuclei and altered microvilli, ranging from short and disorganized to absent. The induction of SPIB expression and phenotypic appearance were consistent with previous analyses (74–76). The differences in the levels of SPIB expression in each model following RANKL treatment likely reflect the retention of cellular programing from the original biopsy specimen source, as noted above. Importantly, the expression of other cellular markers was not significantly altered with the use of RANKL (Table 2 and Fig. S1), yet important differences between models were noted. For example, the expected higher percentage of goblet cells in the colon-derived models (77, 78) was evident at 48 h of differentiation treatment compared to the ileum- and cecum-derived models (Fig. S1), but the increased percentage in the colon was not significantly altered by RANKL treatment. Thus, our results demonstrate that the cellular populations of the HIODEM models are capable of manipulation by ligand treatment to enable studies focused on specific cell types.

To examine the efficacy of the HIODEM models for pathogenesis, we evaluated *Shigella*, Salmonella, and pathogenic E. coli infection in models derived from the terminal ileum, cecum, and colon. Overall, we detected tissue tropism, pathogen targeting of specific cell types, and interesting infection dynamics by using models derived from the natural sites of infection. For *Shigella* (Fig. 4 and Fig. S2), adherence was highest in the cecum and colon, with the rates of both comparable to adherence rates with polarized T84 cells (26). Apical surface adherence was facilitated by *Shigella* adherence factors expressed under the *in vivo*-like culture conditions and replicates previous analyses with both the colon and cecum models (23, 24, 26). Meanwhile, *Shigella* invasion was the highest in colon following treatment with RANKL to induce the presence of M cells. To confirm our data, we repeated analyses with another patient-derived colon model and detected an approximately 10-fold increase in recovery, in which the DAPT plus RANKL treatment increased S. flexneri colonic invasion relative to DAPT treatment (data not shown). Overall, both sets of invasion rates were reduced relative to rates seen in nonpolarized HeLa cells (69) or direct basolateral administration of polarized T84 cells (67, 79), but these models promote almost complete infection of the host cells and bypass important steps during the infection process. Since the HIODEM model is composed of various cell types, we only expect *Shigella* infection of the enterocytes. Our results agree with two recent publications using variations of the organoid-derived monolayer model that also verified the colonic tropism and the basolateral entry preference for *Shigella* invasion (54, 55). In this study, microscopy analyses indicated bacterial transit of M cells, which complements both previous confocal microscopy analyses indicating *Shigella* translocation of cecum-derived organoids following apical administration (23) and the invasion data rates obtained in the analyses described here. Furthermore, infection with the virulence plasmid-cured strain BS103 resulted in minimal recovery of the bacteria following gentamicin treatment, confirming that wild-type 2457T recoveries in the presence of gentamicin were due to bacterial invasion of the model. In all, the bacterial culture conditions, apical administration of the inoculum, and use of RANKL to promote M cell differentiation enables human-specific conditions to replicate the natural *Shigella* infection process in the laboratory setting.

In the literature, both *S*. Typhi and *S*. Typhimurium have been reported to infect the ileum (80–84) but result in differing pathologies, either systemic infection in the case *S*. Typhi or localized gastroenteritis in the case of *S*. Typhimurium. To explore this phenomenon in the HIODEM model, we analyzed infection of both serotypes in the ileum, cecum, and colon (Fig. 5 and Fig. S3). As with the *Shigella* analyses, we used bacterial culture conditions to induce virulence factor expression. Thus, *S*. Typhi was cultured with high-salt media and static growth as previously described (33, 85), and the same protocol was used for *S*. Typhimurium to maintain similar growth conditions. The infection analyses demonstrated the surprising result that *S*. Typhi preferentially infected the cecum while *S*. Typhimurium preferentially infected the ileum and caused more cellular destruction relative to *S*. Typhi. Furthermore, treatment of ileum-derived HIODEM monolayers with RANKL to induce M cell differentiation did not enhance *S*. Typhi invasion, which contrasts with *S*. Typhimurium observations (75) but is consistent with our previous analyses that *S*. Typhi invades human biopsy specimens via the apical surface of enterocytes in which no bacterial associations with M cells were detected (33). Thus, the HIODEM model reproduced the serovar-specific differences that are expected given the different GI pathologies associated with each pathogen while also identifying a unique cecum-specific infection pattern for *S*. Typhi. Reports of *S*. Typhi infection or damage to the cecum have been documented in the literature. Colonoscopic evaluations of patients with typhoid fever have found intestinal lesions in the terminal ileum in all patients tested, with additional lesions identified by the ileocecal valve and ascending colon (86). Furthermore, perforations of the ileocecum or lower gastrointestinal bleeding associated with the cecal artery have been reported in patients with *S*. Typhi infection (87, 88). Therefore, the HIODEM system offers opportunities to understand key differences between *S*. Typhi and *S*. Typhimurium, which may correlate with genetic differences of the pathogens (89) and/or epigenetic differences in humans.

Finally, the E. coli pathovar analyses validated the HIODEM system as a human-specific model that recapitulates expected infection patterns. Robust infection of the appropriate site of the GI tract was observed with each pathovar, with comparable rates of adherence and minimal recoveries upon gentamicin treatment to indicate a lack of invasion. The infection patterns and microscopic evaluations are in agreement with previous and recent analyses of organoid monolayer-based systems (11, 56, 57, 72) as well as with established literature regarding actin association/pedestal formation or aggregative adherence patterns (37, 38). As with *Shigella* and Salmonella analyses, the future applications of the HIODEM model system with all E. coli pathotypes are broad and expected to provide key insights into human-specific pathology, including infection analyses in other segments of the GI tract for each pathovar.

In summary, we have provided reproducible infection analyses of six bacterial pathogens spanning three genera, in which unique observations have already been provided and infection data have been verified with electron microscopic analyses. This model and approach can be applied to additional enteric pathogens or bacteria representing the human microbiota. Coupled with *in vivo-*like bacterial culture conditions, the HIODEM model offers one of the most human-specific infection analyses that can be performed in the laboratory setting to further our understanding of host-microbe interactions and hopefully help lead to the discovery of novel vaccines and therapeutics.

## MATERIALS AND METHODS

### Human subject research, IRB approval, and biopsy specimen collection.

Human sample collection was approved by Institutional Review Board (IRB) protocols 2014P002001 and 2015P001908 of the Massachusetts General Hospital, Boston, MA. Donor tissue was obtained from consenting patients undergoing medically required colonoscopies or surgical resections, as determined by a licensed physician. All subjects provided written informed consent for samples to be used for research purposes.

### Organoid culture and monolayer generation.

The protocol has been adapted from previous publications (11, 15, 33). Please refer to the supplemental material for step-by-step instructions. Briefly, stem cells derived from donor biopsy specimens were maintained in Matrigel culture in a 1:1 mixture of intestinal stem cell medium (ISC) plus L-WRN conditioned medium containing the inhibitors Y-27632 and A 83-01. Cells were seeded at a density of 15,000 cells per Matrigel dome and grown for 7 days in culture. Spheres were trypsinized into a single-cell state, seeded onto polyethylene terephthalate (PET) membrane transwell inserts with a 0.4-μm pore size at 1.0 × 10^6^ cells/ml, and incubated in 1:1 stem cell medium–L-WRN medium at 37°C with 5% CO_2_. The culture medium was changed every other day until the cultures reached confluence, as determined by TEER monitoring and microscopic observation. Cells grew and matured for 7 to 10 days, at which time the apical and basolateral media were changed and differentiation reagents were applied for either 24 or 48 h depending on the experiment. Differentiation reagents included 5 μM γ-secretase inhibitor IX (DAPT; Calbiochem) application to the apical surface, which was also combined with 100 or 500 ng/ml the receptor activator of the NF-κB ligand (RANKL; Peprotech) application to the basolateral media where indicated.

### Infection analyses.

For information on infection analyses, please refer to the detailed protocols in the supplemental material.

### TEER.

To assess paracellular permeability, transwell inserts were monitored using a TEER apparatus (World Precision Instruments, Sarasota, FL) per the manufacturer’s instructions (15, 33, 66).

### RT-qPCR analysis.

RNA was extracted from monolayers using TRIzol (ThermoFisher) and a Direct-Zol (Zymo) RNA extraction kit. RNA was treated with on-column DNase. RNA was converted to cDNA using Thermo Scientific Maxima first-strand cDNA synthesis kit. Gene expression quantitation was determined using Sybr green (Perfecta), and the CFX96 real-time PCR detection system (Qiagen, Venlo, Netherlands) was used for gene expression analysis. The relative threshold cycle (ΔΔ*C_T_*) method was used for assessing gene expression relative to the 18S housekeeping reference gene (15, 33). Identification of cell type was based on the following genes: *LGR5* (90–92) for stem cells, *ESE1* (19) and *SIM* (sucrose isomaltase) (93) for enterocytes, *KLF4* (20) and *MUC2* (77) for goblet cells, *SPIB* for M cells (17), *CGA* (94) and *NEUROD* (95) for enteroendocrine cells, *OCLDN* (occludin) (96, 97) for barrier formation, and *SOX9* (98, 99), *SPDEF* (98, 100), and *LYZ* (lysozyme) (101, 102) for Paneth cells.

### Flow cytometry.

After differentiation, large transwells (12-well plates) were trypsinized for 10 min. Cells were subsequently resuspended in cDMEM and kept on ice until staining. Cells were fixed, permeabilized, and stained with a flow cytometry fixation and permeabilization buffer kit as directed by the manufacturer. Briefly, cells were washed twice with 1× phosphate-buffered saline (PBS; Gibco), resuspended in 500 μl of flow cytometry fixation buffer (R&D), and incubated for 10 min at room temperature. Following fixation, cells were permeabilized with 200 μl of flow cytometry permeabilization/wash buffer (R&D) and stained with anti-human ESE1 (Abcam), anti-human KLF4 allophycocyanin-conjugated (R&D System), and anti-human SPIB (Invitrogen) antibodies by incubating for 45 min at 4°C. Afterwards, the excess antibodies were removed by washing the cells with flow cytometry permeabilization/wash buffer and stained with secondary antibodies anti-rabbit IgG1 FITC (Abcam) and anti-mouse IgG2 peridinin chlorophyll protein (BD Bioscience) for 20 min at 4°C. Samples were washed again with flow cytometry permeabilization/wash buffer, fixed in 1% paraformaldehyde in 1× PBS (Gibco), and acquired with BD FACSCalibur flow cytometer. Analysis was performed with BD Biosciences software. Enterocytes (ESE1^+^), M cells (SPIB^+^), and goblet cells (KLF4^+^) were gated among live cells based on forward and side scatter parameters.

### Electron microscopy.

For transmission electron microscopy (TEM) analysis, samples were fixed in 2% paraformaldehyde–2.5% glutaraldehyde in 0.1 M sodium cacodylate followed by mounting on grids and imaged using a transmission electron microscope (JEOL, Peabody, MA). For scanning electron microscopy (SEM) analysis, HIODEM monolayers were fixed in 0.5× Karnovsky fixative (Newcomer Supply) and subsequently stored in 1× PBS at 4°C. All sample processing occurred at the Massachusetts Eye and Ear Infirmary core facility. All SEM imaging was performed at the Harvard University Center for Nanoscale Systems (CNS) using a FESEM Supra55VP microscope.

### Cytokine analysis.

Quantification of secreted interleukin 8 (IL-8) was conducted using R&D Systems human CXCL8/IL-8 DuoSet enzyme-linked immunosorbent assay per the manufacturer’s instructions (103).

### LDH assay.

Apical supernatants were assessed for LDH release using a Promega Cytox kit (Promega, Madison, WI) according to the manufacturer’s instructions (33).

### Immunostaining.

For immunostaining, monolayers were fixed in 4% paraformaldehyde at room temperature for 15 min, followed by storage in 70% ethanol at 4°C until paraffin embedding (33). Embedding and sectioning were performed by the Specialized Histopathology Core of Massachusetts General Hospital. Prior to staining, sections were deparaffinized using xylene with gradual rehydration in decreasing concentrations of ethanol. Sections were blocked using 0.4% goat and donkey serum in 0.04% Triton X-100 in PBS. Sections were stained using the antibodies against actin (3700S; Cell Signaling Technologies), Mucin 2 (sc-13312; Santa Cruz Technologies) (33), Salmonella (8209-4006; Bio-Rad), and E. coli (ab137967; kind gift of Deepak V. K. Kumar; Abcam). Fluorescently conjugated secondary monoclonal antibodies (Alexa Fluor 488- and 555-conjugated antibody series against mouse, rabbit, or goat from Life Technologies) were used for detection. Nuclei were counterstained with 6-diamidino-2-phenylindole (DAPI). Samples were imaged using a Nikon A1SiR confocal microscope.
