# Supplementary material for: A Versatile Human Intestinal Organoid-Derived Epithelial Monolayer Model for the Study of Enteric Pathogens
Source: Microbiol Spectr. 2021 Jun 9;9(1):10.1128/spectrum.00003-21. doi: 10.1128/spectrum.00003-21 (PMC8552518; doi:10.1128/spectrum.00003-21)
Supplement: SUPPLEMENTAL FILE 1 — Fig. S1 and S2; supplemental methods. Download SPECTRUM00003-21_Supp_1_seq8.pdf, PDF file, 1.2 MB [file spectrum00003-21_supp_1_seq8.pdf]

Supplemental Figure S1. Additional flow cytometry data.

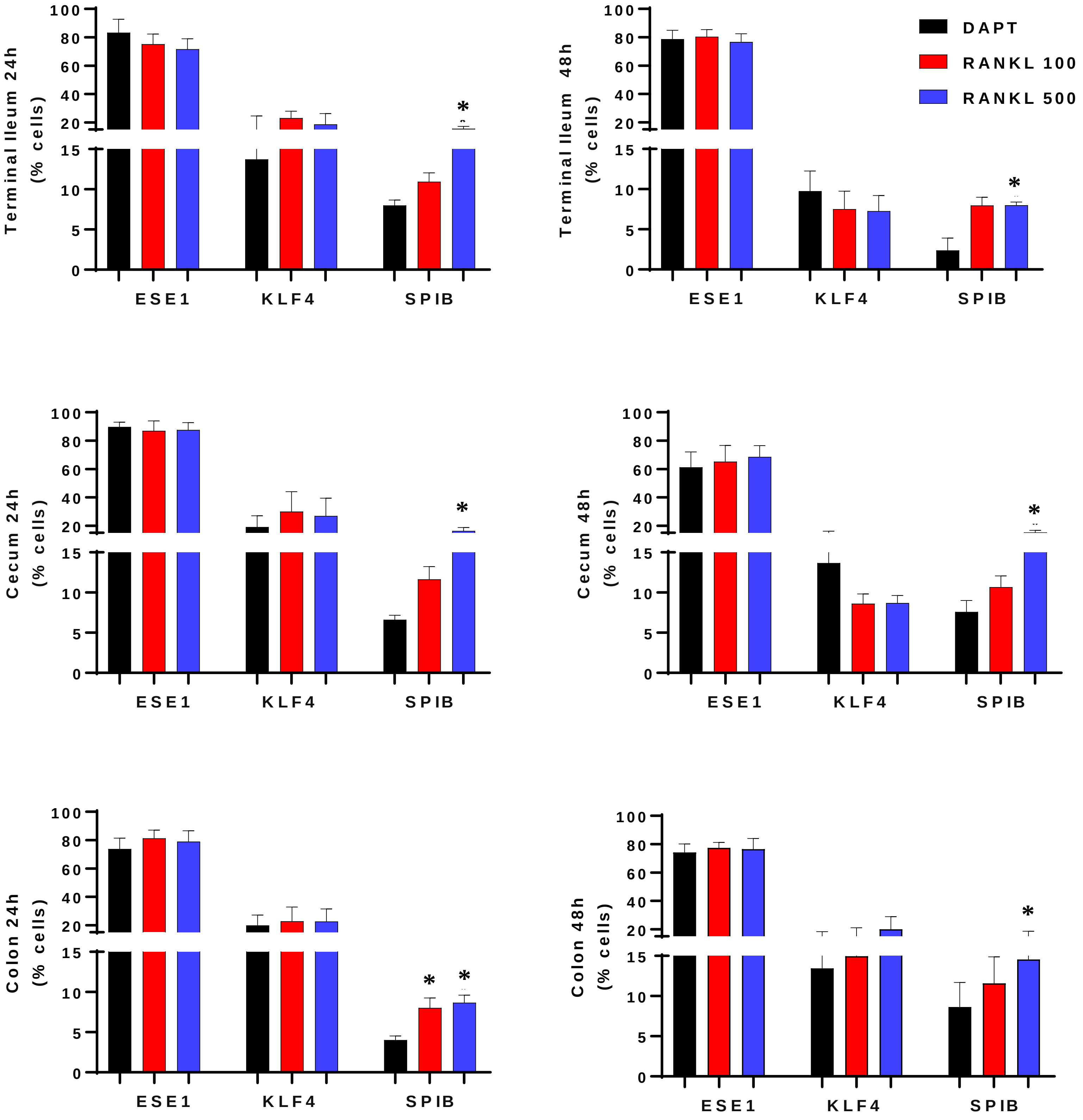

Legend: The percentage of each cell type (enterocytes (ESE1), goblet cells (KLF4), and M cells (SPIB)) detected by flow cytometry for the terminal ileum, cecum, and colon. The data represent the average percentage +/- SEM of each cell population at 24- and 48-hours following differentiation, in which treatments included apical DAPT (black bars), or a combination of apical DAPT with basolateral physiological 100 ng/ml RANKL treatment (red bars) and supraphysiological 500ng/mL (blue bars). Statistical significance was determined with a one-way ANOVA (paired Friedman test) for the samples treated with RANKL versus the DAPT only treatment of the matched originating tissue (* = $p<0.05$). Please note that the SPIB data corresponds to the data presented in Figure 1.

Supplemental Figure S2. Additional analysis of *Shigella flexneri* HIODEM infection.

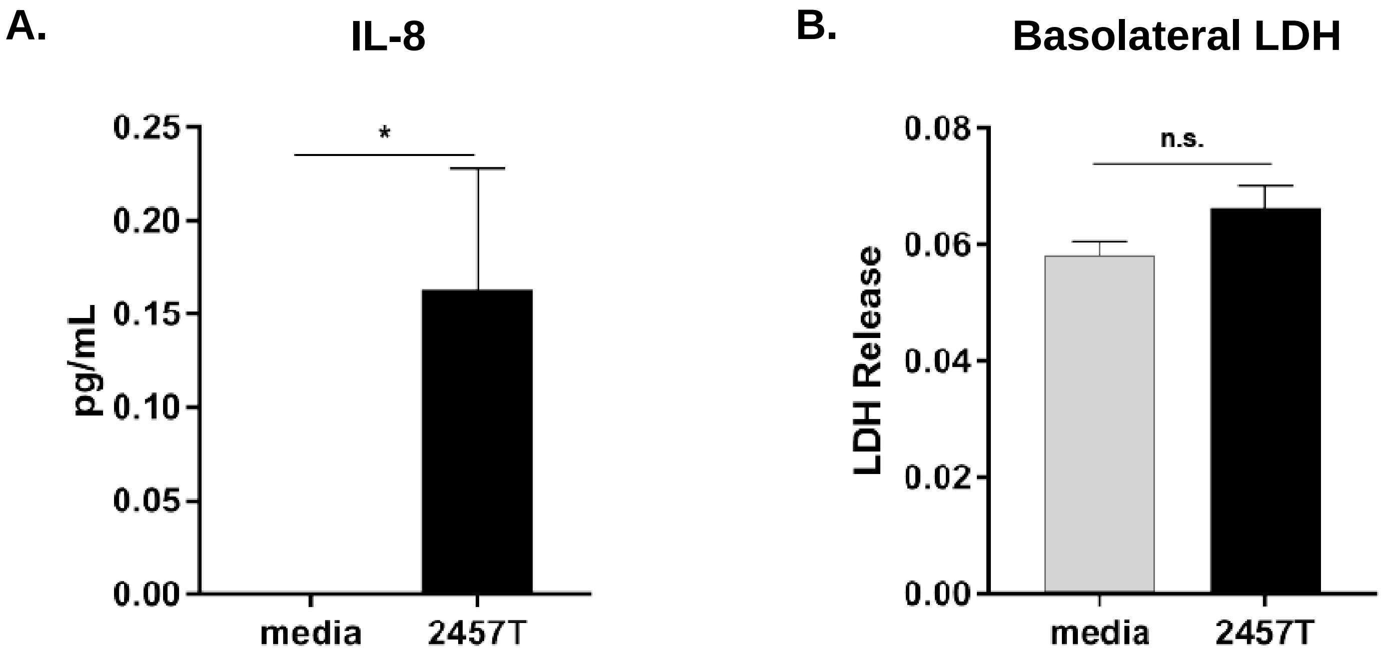

Legend:

A. Colon-derived HIODEM monolayers were treated with DAPT + RANKL. Uninfected monolayers (media) or infected with *S. flexneri* demonstrate significant secretion of IL-8 after infection. Statistical significance was determined by the Student's t-test, * = $p \leq 0.05$.

B. No significant increase in cell death as evaluated by LDH-release was detected following infection of colon-derived HIODEM monolayers treated with DAPT + RANKL.

Supplemental Figure S3. Additional microscopy analysis of *Salmonella* HIODEM infection.

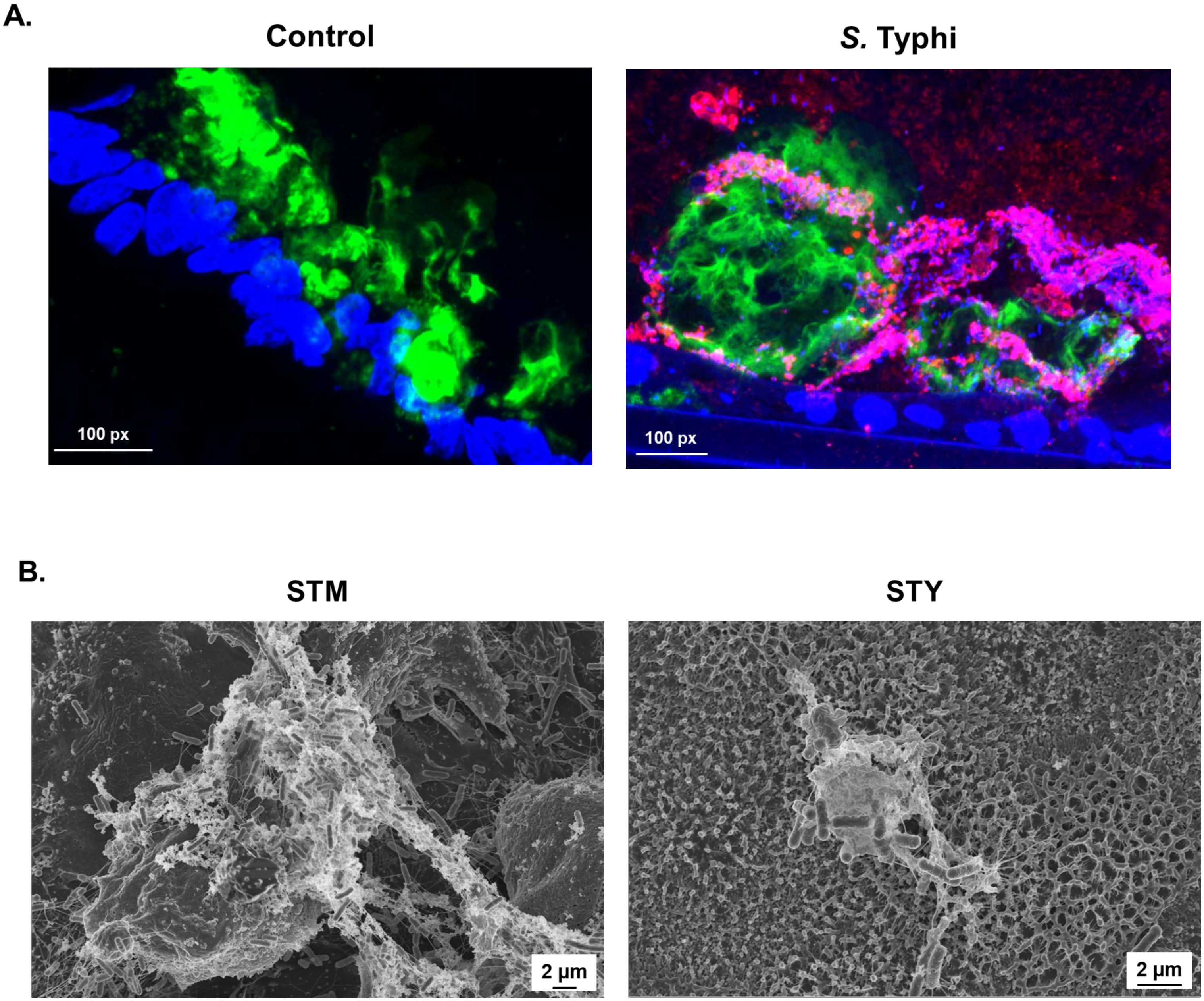

Legend:

A. Confocal immunofluorescence analysis of *S.* Typhi HIODEM infection. Images of control (left) or *S.* Typhi infection (right) of ileum-derived HIDOEM. Samples were stained with DAPI to visualize cellular DNA (blue), α-Muc2 to identify the mucus barrier secreted from goblet cells (green), and α-*Salmonella* to visualize the infecting population (red). Colocalization of *Salmonella* and DAPI results in the bacteria appearing purple.

B. Scanning electron micrographs of STM- or STY-infected ileum HIODEMs. As demonstrated in Figure 5, STM infection led to significant destruction of the barrier, while STY infection did not affect the barrier and the bacteria associated with microvilli on the apical surface of the enterocytes. 2 μm scale bars are indicated.

Supplemental Methods

1. <u>Human Subjects Research, Recruitment, and Institutional Approval</u>

Important: Appropriate Institutional Review Board and biosafety approval are required prior to beginning any of the presented protocols. For donors enrolled in our study, the following exclusion and inclusion criteria were applied:

<u>Inclusion Criteria</u>

- Male or female children and adults aged 14 to 80 years.
- Scheduled to undergo diagnostic colonoscopy at Massachusetts General Hospital
- Good general health with no chronic medical conditions

<u>Exclusion Criteria</u>

- Unable or unwilling to provide written informed consent
- Known diagnosis of autoimmune or other chronic medical conditions (including, but not limited to diabetes, lupus, etc.)
- Pregnant women
- Any medical condition that would increase the risk of bleeding or perforation from a gastrointestinal biopsy
- Irritable Bowel Syndrome
- Any history of polyps

Note. The authors recommend use of biopsies for isolation of intestinal stem cells. An alternative tissue source is resected tissue; however, additional exclusion criteria and restrictions apply. In the case of tumor resection, tissue used for stem cell isolation must be at least 10 centimeters from the tumor location with clean margins as determined by a pathologist.

For the purposes of discussion, all donor-derived cultures will be referred to as organoids. The authors are aware of specific terminology for ileum-derived organoids (enteroids) or colon-derived organoids (colonoids) and wish to keep the text all-inclusive as these protocols apply to cultures from the ileum, cecum, and colon.

2. <u>Isolation of Donor Stem Cells</u>

Protocol adapted from References (1-3). Please see reagent preparation below.

Note. To ensure enough starting material for isolation of stem cells, the authors recommend the use of large forceps for biopsy collection during endoscopy.

2.1. Collect four biopsies (each approximately 4 x 7 mm) from the intestine into a conical tube filled halfway with 1X cDMEM/F12. Keep the tube on ice after collection and during transport to the biological safety hood.

2.2. In a biological safety hood, transfer biopsies to a sterile petri dish containing PBS + penicillin/streptomycin (P/S). Allow biopsies to equilibrate for 5 minutes on ice.

2.3. Transfer biopsies to a 15 mL conical tube containing 10 mL dissociation buffer (DB). Incubate samples buried in ice for 30 minutes.

2.3.1. While incubating, label five 15 mL conical tubes with sample ID and fractions 1-5.

2.4. Remove the biopsy tube from ice. Shake the tissue vigorously for at least 30 seconds (approximately 120 shakes per minute).

2.5. Working around the biopsies floating in the buffer, collect the supernatant and transfer to the tube labeled “fraction 1”.

2.6. Add 1 mL of sterile FBS to the “fraction 1” tube and invert 2-3 times to mix and store on ice.

2.7. Add 10 mL of fresh DB to the biopsies. Invert the tube to make sure all biopsies are submerged. Incubate on ice for 30 minutes.

2.8. Repeat the process until all five fractions are collected.

2.9. Spin all fractions at 400 RPM (100 x *g*) for 10 minutes.

2.10. Remove the supernatants and resuspend the pellets in 500 μL cDMEM/F12 (see reagents section for media preparation instructions), transferring the samples from a 15 mL conical tube to a sterile 1.5 mL microcentrifuge tube.

2.11. Evaluate the crypt purity and number using a hemocytometer

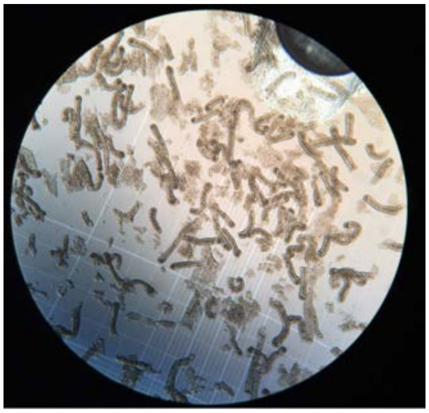

A sample image of crypt examination with a hemocytometer from an ileum biopsy collection.

2.12. Combine the fractions with the most crypts into a single 1.5 mL tube.

Note: Usually fractions 2 to 4 are enriched in crypts.

2.13. In a benchtop microcentrifuge spin at 1200 RPM (140 x *g*) for 5 minutes at 4°C.

2.14. Carefully remove and discard the cell supernatant without disrupting the cell pellet. Subsequently, add about 230 µL of Matrigel on ice.

Note: The Matrigel will solidify when warmed to room temperature. Keep on ice while working. The authors recommend use of Matrigel low growth factors with a protein content between 9.0-9.5 mg/ml and endotoxin unit <1.5.

2.15. Keeping the tube on ice, pipette up and down to mix the Matrigel-cell solution.

2.16. Gently and quickly, pipette 11.5 µL drops of the Matrigel-cell suspension into 2 wells of a 6-well plate. Approximately ten droplets will fit each well of a 6-well plate.

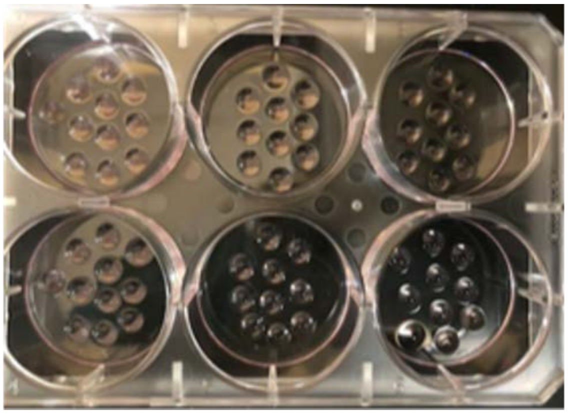

A sample image of adding the Matrigel-cell suspension drops to each plate.

2.17. Incubate at 37°C for 10 minutes.

2.18. While the Matrigel is setting, prepare 1:1 mixture of intestinal stem cell media (ISC) + L-WRN conditioned media containing the inhibitors Y-27632 and A 83-01 (1:1+A/Y medium).

2.19. Add 2.5 mL 1:1+A/Y to cells and return to the incubator.

2.20. After 24-48 hours, monitor for formation of organoids and proceed to **3.1 Maintenance** and **3.2 Splitting cells**.

3. <u>Organoid Culture</u>

3.1. Maintenance

3.1.1. Evaluate the organoids under the microscope. Ensure organoid are not contaminated by bacterial or fungal growth. *Note: The authors recommend preparation of stock LWRN conditioned medium at least every three months if stored at -20°C. Furthermore, thawed aliquots should be used within 4 days of thawing. In our experience, the shape of the organoids*

varies from spheres to glandular with crypt projections on the surface, depending on donor's samples.

3.1.2. Replace culture media with 1:1+A/Y every 2 to 3 days.

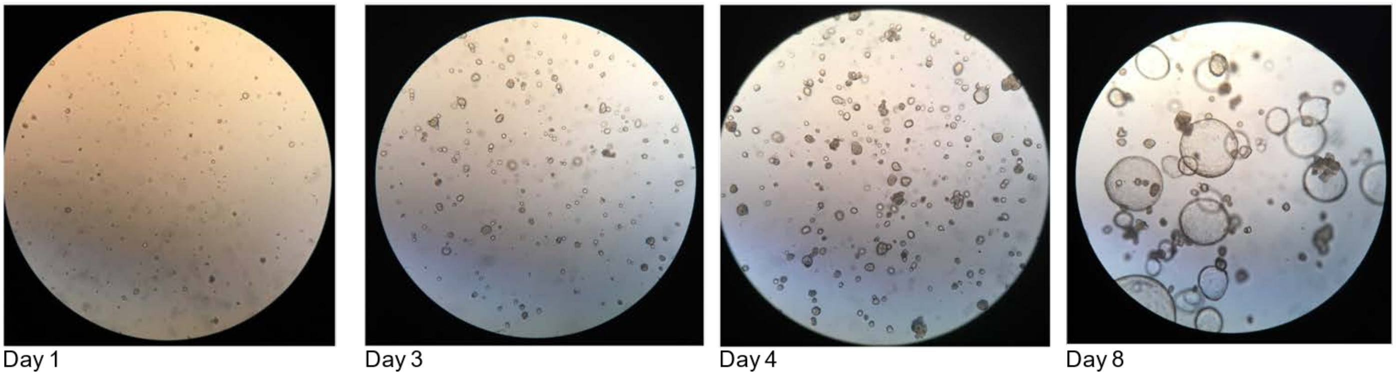

A sample set of images of organoid maturation over time.

3.2. Splitting Cells

3.2.1. Prepare an ice bucket with DB and a 15 mL conical tube for sample collection.

3.2.2. Working in a biological safety cabinet, remove media from organoids. Add 1 mL DB to each well of a six well plate that contains organoids.

3.2.3. Pipette up and down and gently scrape to dislodge the Matrigel domes from the plate while simultaneously dissociating the organoids from the matrix.

3.2.4. Transfer to a 15 mL conical tube. One to three wells from a 6 plate well can be collected per 15 mL conical. More wells can lead to too much Matrigel and can be difficult to isolate the cells.

3.2.5. Rinse the wells with additional 1 mL DB to collect all cells and transfer to the same 15 mL conical tube.

3.2.6. Bring the total volume up to 12 mL of DB and shake by hand vigorously (approximately 120 shakes per minute) to break down the Matrigel.

3.2.7. Centrifuge at 1000 RPM (200 x *g*) for 10 minutes.

3.2.8. Carefully remove the dissociation buffer down to 500 µL.

3.2.9. Add 500 µL of warm (37°C) trypsin.

3.2.10. Using a 21-gauge x 25 mm needle and 5 mL syringe, gently pipette the pellet and trypsin suspension up and down for 2 minutes.

3.2.11. Add 4.5 mL of cDMEM/F12 to the trypsin-cell mixture.

3.2.12. Filter the cells using a 70 µm nylon mesh.

3.2.13. Pellet by centrifugation at 1000 RPM (200 x *g*) for 10 minutes.

3.2.14. Resuspend the pellet in 1:1 mixture of intestinal stem cell media (ISC) + L-WRN conditioned media containing the inhibitor Y-27632 (1:1+Y; usually 1-3mL) and evaluate the cell count and dissociation status using a hemocytometer or cell counter. *Note: At this point save the number of cells that will be used for the next step.*

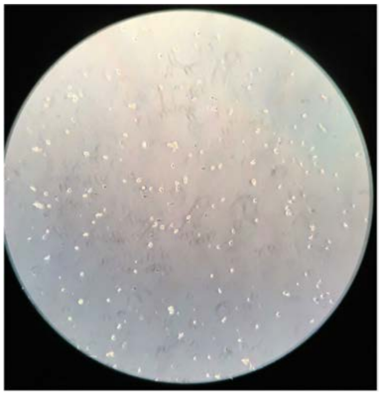

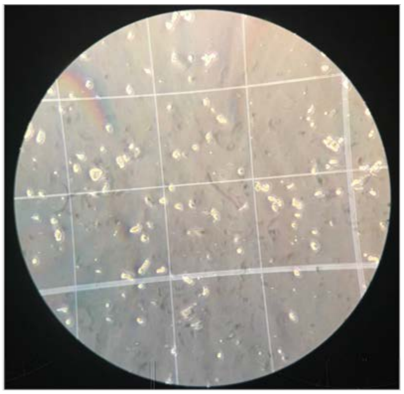

Sample images of single cells prior to seeding.

3.2.15. Continue to the next protocols to seed the cells for organoids maintenance (**3.2.15.1**) or to seed monolayers (**3.2.15.2**).

3.2.15.1. Seeding for organoids subculture

3.2.15.1.1. Resuspend the cell pellets in 1mL of 1:1+A/Y.

3.2.15.1.2. Transfer to a 1.5 mL microcentrifuge tube.

3.2.15.1.3. Centrifuge at 1200 RPM (140 x *g*) for 5 minutes in a 4°C refrigerated microcentrifuge.

3.2.15.1.4. While the cells are spinning, determine the amount of Matrigel needed for culture. In our experience, plating 15,000 cells per dome in a 6-well plate guarantees a robust sub-culture of the organoids.

3.2.15.1.5. Discard the supernatant by pipetting slowly to avoid disturbing the cell pellet.

3.2.15.1.6. Resuspend the cell pellet in the amount of Matrigel determined in step 3.2.12.1.4

3.2.15.1.7. Plate 11.5 µL organoids in a six-well plate, fitting 10 Matrigel domes per well.

3.2.15.1.8. Incubate the plates for 10 minutes at 37°C.

3.2.15.1.9. Cover the domes with 2.5 mL 1:1+A/Y per well. Incubate at 37°C with 5% CO_2 until feeding 2 to 3 days later. Media should be changed on a regular schedule to prevent drop in stem cell growth factors and premature differentiation. Organoids have shown to be transcriptionally stable up to passage 25[1].

3.2.15.2. Seeding for monolayer generation

3.2.15.2.1. Resuspend cells in 1:1 + Y at 1 x 10^6 cells/mL.

Note. At this step, the A 83-01 inhibitor is no longer required for culture. In our experience A 83-01 does not favor the attachment of the cells to the transwell membrane. Different transwells can be used; the authors best results have been obtained using 0.4 µm pore size polyester transwell.

3.2.15.2.2. Add 400 µL to the apical chamber of transwell inserts (surface area 1.12 cm^2) or 100 µL to the apical chamber of the transwell inserts (surface area .33cm^2). Distribute the cell suspension uniformly by gently moving the plates back and forward twice. Do not apply rotating movement.

3.2.15.2.3. Add 1 mL 1:1+Y basolateral chamber large transwells or 600 µL 1:1+Y basolateral chamber small transwells.

3.2.15.2.4. Carefully transfer the plates to an incubator at 37°C with 5% CO_2.

3.2.15.2.5. Change media every 2 to 3 days and monitor changes in transepithelial electrical resistance (TEER). *Note: The transwell manufacturer (Corning) recommends removing medium from the basolateral chamber, then apical before filling the apical chamber first, and basolateral second. Please consult the manufacturer's instructions for complete details.*

3.2.15.2.6. After 7 to 10 days in culture, TEER values should stabilize and reach a value in the appropriate range for the specific intestinal segment derived organoids (see **Table 1** of main text).

3.2.15.2.7. Begin differentiation of the monolayer by treating the apical surface with cDMEM/F12 + 5µM N-[N-(3,5-Difluorophenacetyl)-L-alanyl]-S-phenyl-glycine t-butyl ester (DAPT) (1, 3, 4). Replenish the basolateral medium with 1:1 (+ Y optional). Incubate for 48 hours. Monolayers are now ready for experimentation. *Note: At this point Y inhibitor can be omitted depending on the experimental design requirements.*

3.2.15.2.7.1. Optional: treatment of the basolateral pole with 100 ng/mL receptor activator of nuclear factor kappa-B ligand (RANKL (5)) will promote M cell differentiation. The treatment occurs concurrently with the apical DAPT treatment.

4. **Pathogen Infection**

4.1. *Shigella* species

The authors would like to note that the growth conditions are optimized for S. flexneri to maximize virulence by culturing the bacteria in media supplemented with both bile salts and glucose, which represent physiological stimuli previously shown to be significant in promoting adherence and invasion (6-9).

4.1.1. **[Bacteria]** Maintain *Shigella* stock cultures on TSB + Congo red agar at 4°C. Restreak plates every two weeks, and restreak from a glycerol stock monthly. The colony morphology should be small, glossy, round, and red. The red color indicates expression of the virulence plasmid when grown at 37°C (10, 11). Discard plates with numerous white colonies and ensure that stock plates are grown in an incubator maintaining at least 37°C as expression of the virulence plasmid is temperature dependent (12).

4.1.2. **[Bacteria]** One day prior to the experiment (24 hours into monolayer differentiation), inoculate 3 mL of TSB with a single, well-isolated colony of *Shigella* from a stock plate.

4.1.3. **[Bacteria]** Incubate the culture at 37°C overnight, with shaking at 225 RPM.

4.1.4. **[Bacteria]** The next day, make a 1:50 subculture into TSB + 0.4% bile salts.

Note*. Bacterial growth conditions are critical to evaluating invasion. In previous studies, the authors have shown that Shigella responds to bile salts with induced virulence (6-9), aspects of which also require the presence of glucose (8, 9), that includes increased epithelial cell attachment, virulence gene expression, and epithelial cell invasion. TSB growth medium contains added glucose relative to LB (8), which is maintained in the overnight and secondary cultures and further supplemented with 0.4% w/v bile salts in the secondary culture.*

4.1.5. **[Bacteria]** Incubate the culture at 37°C with shaking at 225 RPM until the culture reaches an optical density (OD_{600}) of 0.7.

4.1.6. **[Monolayers]** While the bacteria are growing, change the media on the cells. Wash the basolateral chamber twice with PBS and replace with 1X DMEM.

Note*: We used DMEM without phenol red for our monolayer experiments to enable the use of FITC-dextran as a secondary measure of paracellular transport.*

4.1.7. **[Monolayers]** Remove the apical media and gently wash twice with warm (37°C) PBS. Replace the PBS with 1X DMEM.

Note: Washing removes trace antibiotics at the expense of removing the mucus barrier. The authors washed twice prior to Shigella infection to remove any residual culture antibiotics as well as the mucus barrier.

4.1.8. **[Bacteria]** Determine the OD_{600} of the culture.

4.1.8.1. The culture density should be at least 0.7, which indicates the bacteria are in the early exponential phase of growth.

4.1.9. **[Bacteria]** Prepare the inoculum by standardizing the culture to an $0D_{600}$ of 0.35 and diluting accordingly. For example, divide 0.35/0.7 per 1 mL, which results in using 500 µl of the culture and adding 500 µl warm (37°C) PBS. This standardization process corresponds to a final infecting titer of approximately 1 x 10^8 CFU/mL.

4.1.10. **[Bacteria]** Transfer the standardized culture (per 1 mL) to a 1.5 mL microcentrifuge tube.

4.1.11. **[Bacteria]** Pellet by centrifugation at 12,000 RPM (12,400 x *g*) for 2 minutes.

4.1.12. **[Bacteria]** Remove the supernatant and wash with 1 mL of warm (37°C) PBS.

4.1.13. **[Bacteria]** Repeat the wash step 2 times.

4.1.14. **[Bacteria]** Resuspend the pellet in 1 mL of warm (37°C) 1X DMEM.

4.1.15. **[Monolayers]** Remove the plate from the incubator and record the baseline TEER values.

4.1.16. **[Monolayers + Bacteria]** Remove the apical medium and replace with 40 µL bacteria inoculum or control medium.

4.1.17. Return the plate to the incubator for 2 hours for colon infection or 3 hours for terminal ileum or cecum infection.

Note: During these assays, the authors observed that apical invasion of ileum or cecum-derived HIODEM cells was observed after 3h of infection. By contrast, RANKL-differentiated, colonic-derived HIODEM cells had intracellular bacteria detected as early as two hours after infection. This variable is adjustable depending on the experimental question.

4.1.18. Plate the inoculum to verify the infecting bacterial titer added to the cells.

4.2. For the adherence assay, record the TEER and remove the apical and basolateral supernatants.

Note: Supernatants can be saved for assessment of cytokines or cell viability.

4.2.1. Wash the wells 3 times with warm (37°C) PBS.

4.2.2. Lyse the monolayers in 200 µL warm (37°C), sterile 1% Triton X-100 in PBS. Incubate at RT on a shaker for 30 minutes.

4.2.3. Add an additional 800 µL warm (37°C), sterile 1% Triton X-100 in PBS to each transwell and scrape to lyse the cells. Pipette up and down and break the transwell to ensure complete lysis of cells.

4.2.4. Dilute lysate in PBS. Plate 100 µL of the 10^{-2} through 10^{-7} dilutions to ensure countable colony numbers (ranging from 30 to 300).

4.2.5. Incubate at 37°C overnight.

4.2.6. The next day, count the colonies to determine bacterial adherence rates.

4.2.7. To calculate % adherence: Divide the total adherent bacteria (accounting for dilution) by the total infecting titer and multiply by 100.

4.3. For the invasion assay, remove the apical and basolateral supernatants.

Note: supernatants can be saved for assessment of cytokines or cell viability.

4.3.1.Wash the wells 3 times with warm (37°C) PBS.

4.3.2.Replace the apical and basolateral chambers with DMEM containing gentamicin (50 µg/mL). Incubate at 37°C for 3 hours.

4.3.3.Wash the wells 3 times with warm (37°C) PBS.

4.3.4.Lyse the monolayers in 200 µL warm (37°C), sterile 1% Triton X-100 in PBS. Incubate at RT on a shaker for 30 minutes.

4.3.5. Add an additional 800 µL warm (37°C), sterile 1% Triton X-100 in PBS to each transwell and scrape to lyse the cells. Pipette up and down and break the transwell to ensure complete lysis of cells.

4.3.6.Dilute lysate in PBS. Plate 500 µL of the undilute lysate and 100 µL of the 10^{-1} and 10^{-3} dilutions to ensure countable colonies (30 to 300).

4.3.7.Incubate at 37°C overnight

4.3.8.The next day, count the colonies to determine bacterial invasion rates.

4.3.9.To calculate % invasion: Divide the total intracellular bacteria (accounting for dilution) by the total infecting titer and multiply by 100.

4.4. *Salmonella* species

The authors would like to note that the growth conditions are optimized for Salmonella enterica serovar Typhi to maximize expression of SPI-1 (static growth (3)) and the Vi antigen (high-salt media (3, 13)). To maintain similar conditions, S. Typhimurium is grown using the same protocol.

4.4.1. **[Bacteria]** Maintain *Salmonella* stock cultures LB agar at 4°C. Restreak plates every two weeks, and restreak from a glycerol stock monthly. The colony morphology should be uniform, small, glossy, round and opaque.

4.4.2. **[Bacteria]** 24 hours prior to the experiment (24 hours into monolayer differentiation), inoculate 3 mL of LB Miller with a single, well-isolated colony of *Salmonella* from a stock plate.

Note: LB Miller contains 10 grams NaCl per L of media. ***The*** *high salt concentration is required for expression of the Vi antigen (13).*

4.4.3. **[Bacteria]** Incubate the culture at 37°C without shaking for 4 to 6 hours.

4.4.4. **[Bacteria]** Afterwards, make a 1:50 subculture into 10 mL LB Miller.

4.4.5. **[Bacteria]** Incubate the culture at 37°C overnight without shaking.

4.4.6. **[Monolayers]** The next day, change the media on the monolayers. Wash the basolateral chamber twice with PBS and replace with 1X DMEM.

Note: We used 1X DMEM without phenol red for our monolayer experiments to enable the use of FITC-dextran as a secondary measure of paracellular transport.

4.4.7. **[Monolayers]** Remove the apical media and gently wash once with warm PBS. Replace the PBS with 1X DMEM. Incubate 3 hours at 37°C.

Note*: Washing removes trace antibiotics at the expense of removing the mucus barrier. To help preserve the mucus, remove of the apical media completely, wash once with PBS and replace with media. A single wash step reduces the amount of antibiotic while maintaining the mucus barrier*.

4.4.8. **[Bacteria]** Near the end of the 3-hour window, determine the OD_{600} of the culture.

4.4.8.1. The culture density should be around 0.5 (acceptable range: 0.4 to 0.6), which indicates the bacteria are in the exponential phase of growth.

4.4.9. **[Bacteria]** Prepare the inoculum by standardizing the culture to an $0D_{600}$ of 0.5 and diluting accordingly. For example, divide 0.5/0.6 per 1 mL, which results in using 833 µl of the culture and adding 167 µl warm (37°C) PBS.

4.4.10. This value corresponds to an infecting titer of 4 x 10^8 CFU/mL.

4.4.11. **[Bacteria]** Transfer the standardized culture (per 1 mL) to a 1.5 mL microcentrifuge tube.

4.4.12. **[Bacteria]** Pellet by centrifugation at 12,000 RPM (12,400 x *g*) for 2 minutes.

4.4.13. **[Bacteria]** Remove the supernatant and wash with 1 mL of warm (37°C) PBS.

4.4.14. **[Bacteria]** Repeat the wash step 2 times.

4.4.15. **[Bacteria]** Resuspend the pellet in 800 µL of warm (37°C) 1X DMEM.

4.4.16. **[Monolayers]** Remove the plate from the incubator and record the baseline TEER values.

4.4.17. **[Monolayers + Bacteria]** Remove 75 µL of the apical medium and replace with 75 µL bacteria inoculum or control medium.

4.4.18. Return the plate to the incubator for 2 hours.

4.5. For the adherence assay, record the TEER and remove the apical and basolateral supernatants.

4.5.1.1. Note: Supernatants can be saved for assessment of cytokines or cell viability.

4.5.2. Wash the wells 3 times with warm (37°C) PBS.

4.5.3. Lyse the monolayers in 200 µL warm (37°C), sterile 1% Triton X-100 in PBS. Incubate at 37°C for 5 minutes.

4.5.4. Add an additional 800 µL warm (37°C), sterile 1% Triton X-100 in PBS to each transwell and scrape to lyse the cells. Pipette up and down and break the transwell membrane while pipetting to ensure complete lysis of cells.

4.5.5. Dilute lysate in PBS. Plate 100 µL of dilutions 10^{-2} through 10^{-7} to ensure countable colony numbers (ranging from 30 to 300).

4.5.6. Incubate at 37°C overnight.

4.5.7. The next day, count the colonies to determine bacterial adherence rates.

4.5.7.1. To calculate % adherence: Divide the total adherent bacteria (accounting for dilution) by the total infecting titer and multiply by 100.

4.6. For the invasion assay, remove the apical and basolateral supernatants.

***Note**: Supernatants can be saved for assessment of cytokines or cell viability.*

4.6.1. Wash the wells 3 times with warm (37°C) PBS.

4.6.2. Replace the apical and basolateral chambers with 1X DMEM containing gentamicin (50µg/mL). Incubate at 37°C for 3 hours.

4.6.3. Wash the wells 3 times with warm (37°C) PBS.

4.6.4. Lyse the monolayers in 200 µL warm (37°C), sterile 1% Triton X-100 in PBS. Incubate at 37°C for 5 minutes.

4.6.5. Add an additional 800 µL warm (37°C), sterile 1% Triton X-100 in PBS to each transwell and scrape to lyse the cells. Pipette up and down and break the transwell to ensure complete lysis of cells.

4.6.6. Dilute lysate in PBS. Plate 500 µL of the undilute lysate and 100 µL of the 10^{-1} to 10^{-3} dilutions to ensure countable colonies (30 to 300).

4.6.7. Incubate at 37°C overnight.

4.6.8. The next day, count the colonies to determine bacterial adherence rates.

4.6.8.1. To calculate % invasion: Divide the total intracellular bacteria (accounting for dilution) by the total infecting titer and multiply by 100.

4.7. *Escherichia coli* pathovars

This protocol has been used for enteropathogenic (*EPEC*), enterohemorrhagic (*EHEC*), *and enteroaggregative (EAEC) E. coli and is provided for reference of the proof-of-concept studies presented.*

4.7.1. **[Bacteria]** Maintain *E. coli* stock cultures on LB agar at 4°C. Restreak plates every two weeks, and restreak from a glycerol stock monthly. The colony morphology should be small, uniform, glossy, round and opaque.

4.7.2. **[Bacteria]** One day prior to the experiment (24 hours into monolayer differentiation), inoculate 3 mL of TSB with a single, well-isolated colony of *E. coli* from a stock plate.

4.7.3. **[Bacteria]** Incubate the culture at 37°C overnight, with shaking at 225 RPM.

4.7.4. **[Bacteria]** The next day, make a 1:50 subculture into TSB.

4.7.5. **[Bacteria]** Incubate the culture at 37°C for 2 hours with shaking at 225 RPM.

4.7.6. **[Monolayers]** While the bacteria are growing, change the media on the cells. Wash the basolateral chamber twice with PBS and replace with 1X DMEM.

4.7.6.1. Note: We used DMEM without phenol red for our monolayer experiments to enable the use of FITC-dextran as a secondary measure of paracellular transport.

4.7.7. **[Monolayers]** Remove the apical media and gently wash twice with warm (37°C) PBS. Replace the PBS with 1X DMEM.

4.7.8. **[Bacteria]** Determine the OD_{600} of the culture.

4.7.8.1. The culture density should be at least 0.7 which indicates the bacteria are in the early exponential phase of growth.

4.7.9. **[Bacteria]** Prepare the inoculum by standardizing the culture to an $0D_{600}$ of 0.35 and diluting accordingly. For example, divide 0.35/0.7 per 1 mL, which results in using 500 µl of the culture and adding 500 µl warm (37°C) PBS.

4.7.10. This standardization process corresponds to a final infecting titer of approximately 1 x 10^8 CFU/mL.

4.7.11. **[Bacteria]** Transfer the standardized culture (per 1 mL) to a 1.5 mL microcentrifuge tube.

4.7.12. **[Bacteria]** Pellet by centrifugation at 12,000 RPM (12,400 x g) for 2 minutes.

4.7.13. **[Bacteria]** Remove the supernatant and wash with 1 mL of warm (37°C) PBS (37°C).

4.7.14. **[Bacteria]** Repeat the wash step 2 times.

4.7.15. **[Bacteria]** Resuspend the pellet in 1mL of warm (37°C), 1X DMEM (37°C).

4.7.16. **[Monolayers]** Remove the plate from the incubator and record the baseline TEER values.

4.7.17. **[Monolayers + Bacteria]** Remove the apical medium and replace with 40 µL bacteria inoculum or control medium.

4.7.18. Return the plate to the incubator for at least two hours.

4.7.19. Plate the inoculum to verify the CFU added to the cells.

4.8. For the adherence assay, record the TEER and remove the apical and basolateral supernatants.

Note: Supernatants can be saved for assessment of cytokines or cell viability.

4.8.1. Wash the wells 3 times with warm (37°C) PBS.

4.8.2. Lyse the monolayers in 200 µL warm (37°C), sterile 1% Triton X-100 in PBS. Incubate at RT on a shaker for 30 minutes.

4.8.3. Add an additional 800 µL warm (37°C), sterile 1% Triton X-100 in PBS to each transwell and scrape to lyse the cells. Pipette up and down and break the transwell to ensure complete lysis of cells.

4.8.4. Dilute lysate in PBS. Plate 100 µL of dilutions 10^{-2} through 10^{-7} to ensure countable colony numbers (ranging from 30 to 300).

4.8.5. Incubate at 37°C overnight.

4.8.6. The next day, count the colonies to determine bacterial adherence rates.

4.8.6.1. To calculate % adherence: Divide the total adherent bacteria (accounting for dilution) by the total infecting titer and multiply by 100.

4.9. For the invasion assay, remove the apical and basolateral supernatants.

Note: Supernatants can be saved for assessment of cytokines or cell viability.

4.9.1. Wash the wells 3 times with warm (37°C) PBS.

4.9.2. Replace the apical and basolateral chambers with DMEM containing gentamicin (50µg/mL). Incubate at 37°C for 3 hours.

4.9.3. Wash the wells 3 times with warm (37°C) PBS.

4.9.4. Lyse the monolayers in 200 µL warm (37°C), sterile 1% Triton X-100 in PBS. Incubate at RT on a shaker for 30 minutes.

4.9.5. Add an additional 800 µL warm (37°C), sterile 1% Triton X-100 in PBS to each transwell and scrape to lyse the cells. Pipette up and down and break the transwell to ensure complete lysis of cells.

4.9.6. Dilute lysate in PBS. Plate 500 µL of the undilute lysate and 100 µL of the 10^{-1} to 10^{-3} lysates to ensure countable colonies (30 to 300).

4.9.7. Incubate at 37°C overnight.

4.9.8. The next day, count the colonies to determine bacterial invasion rates.

4.9.8.1. To calculate % invasion: Divide the total intracellular bacteria (accounting for dilution) by the total infecting titer and multiply by 100.

Reagent Preparation

- **cDMEM/F12: DMEM/F12 COMPLETE**:

  To DMEM/F12 (Gibco cat # 11330-057) add the following:

  •50 mL FBS (aliquots stored at -20°C)

  •5 mL Penicillin/streptomycin (aliquots stored at -20°C)

  •5 mL GlutaMAX (100X) (Gibco 35050-061)

  •5 mL Non-Essential Amino Acids (100X) Gibco 11140-050

  •5 mL Sodium Pyruvate (100X) Gibco 11360-070

- **ISC media (Intestinal Stem Cell Media):**

  Prepare DMEM/F12 COMPLETE as described above and remove 70 mL to clean bottle for general use so that there is 500 mL volume left to add the following components:

  •5 mL N2 Supplement (100X)

  •10 mL B27 Supplement (50X)

  •500 µL EGF (1000X) – 50 µg/mL

  •500 µL Insulin (1000X) – 10mg/mL stock in solution from Sigma

  •500 µL N-acetyl Cysteine (1000X) – 1M stock

  •50 µL Gastrin (10,000X) – 100mM

- **L-WRN:**

100 mL and 200 mL stocks are stored in -20°C for cup to 3 months. It is conditioned media that contains WNT, R-spondin and Noggin (acronym "WRN") (14) and should be stored at 4°C for no more than 4 days.

Note: L-WRN cells were generated by (Stappenbeck laboratory, Washington University School of Medicine, St. Louis, Missouri, USA*) and are available at ATCC #CRL-3276 (14).*

- **DMEM without Phenol Red:**
  Gibco cat# 31053-028

- **PBS P/S:**

To PBS Gibco cat # 10010-049 (without $MgCl_2$ and $CaCl_2$)

•Add 5 mL Pen/Strep

- **Penicillin/Streptomycin (P/S):**

Gibco cat # 15140-122

- **DB: Dissociation buffer:**
  PBS P/S
  EDTA 0.5mM
  DTT 1mM

- **Trypsin:**

Trypsin 0.25% EDTA Gibco cat # 25200-056

- **FBS:**

Purchased as heat inactivated, 50 mL aliquots stored at -20°C

Supplement Stocks:

- **A 83-01:**

Sigma SML0788-5MG

MW = 421.52 g/mol

stock = 5 mM

working stock is 500 µM

- Add 2.3 mL DMSO directly to 5 mg in bottle to reconstitute.
- Dilute this 5 mM stock 1:10 in DMSO for the working stock = 500 µM (1000X)

- **DTT:**

  Sigma MW = 154.25 g/mol

  Stock = 1 M

  - Weigh out 5 grams DTT from Sigma (stored at 4°C)
  - Add 32.42 mL sterile water and filter sterilize using a 0.22 µ filter.
  - Aliquot and store at -20°C.

- **EGF**

Peprotech cat # AF-100-15-1mg

Stock made at 500µg/mL = 10,000X

  - To 1 mg EGF, add 2 mL sterile PBS = 500µg/mL = 10,000X
  - For working stock, dilute 1:10 for 50 µg/mL = 1,000X (1mL stock with 9 mL PBS).
  - Aliquot in 1mLand store at -20°C.

- **Insulin, human**

Sigma cat # I9278-5ml

~10mg/mL In Solution from Sigma.

  - Use at 1:1000; final concentration = 10 µg/mL.

- **N-Acetyl Cysteine**

Sigma cat A9165-5G – [stock] = 500 mM; [final] = 500 µM

  - Weigh out 0.816 g N-acetyl cysteine and resuspend in 10 mL sterile dH_2O.
  - Warm (37°C) in water bath to reconstitute.
  - filter sterilize using a 0.22 µ filter.
  - Aliquot 1 mL to 1.5 mL tubes and freeze at -20°C.

- **Y-27632**

Calbiochem 688000-100 mg; MW = 338.3; stock 10 mM.

  - Add 29.56 mL dH_2O to 100mg powder.
  - Aliquot to 1.5 mL tube and store at -20°C.

- **DAPT**

Calbiochem 565784-10mg γ-secretase inhibitor IX

Purchased in solution at 25 mM.

Stock concentration is 5 mM

- o dilute 1:5 in DMSO
- o aliquot and store at -20°C.

- o **RANKL**

Peprotech AF-310-01 Animal-Free Recombinant Human sRANK Ligand (*E. coli* derived)

Stock concentration is 100 µg/ml

- o Reconstitute in sterile nuclease-free water
- o aliquot and store at -20°C.

References

1. **Senger S, Ingano L, Friere R, Anselmo A, Zhu W, Sadreyev R, Walker A, Fasano A.** 2018. Human fetal-derived enterospheres provide insights on intestinal development and a novel model to study Necrotizing Enterocolitis (NEC). Cellular and Molecular Gastroenterology and Hepatology **5:**549-568.

2. **VanDussen KL, Marinshaw JM, Shaikh N, Miyoshi H, Moon C, Tarr PI, Ciorba MA, Stappenbeck TS.** 2015. Development of an enhanced human gastrointestinal epithelial culture system to facilitate patient-based assays. Gut **64:**911-920.

3. **Nickerson KP, Senger S, Zhang Y, Lima R, Patel S, Ingano L, Flavahan WA, Kumar DKV, Fraser CM, Faherty CS, Sztein MB, Fiorentino M, Fasano A.** 2018. *Salmonella* Typhi Colonization Provokes Extensive Transcriptional Changes Aimed at Evading Host Mucosal Immune Defense During Early Infection of Human Intestinal Tissue. EBioMedicine **pii: S2352-3964:**30133-30136.

4. **Wang Y, Kim R, Gunasekara DB, Reed MI, DiSalvo M, Nguyen DL, Bultman SJ, Sims CE, Magness ST, Allbritton NL.** 2018. Formation of Human Colonic Crypt Array by Application of Chemical Gradients Across a Shaped Epithelial Monolayer. Cell Mol Gastroenterol Hepatol **5:**113-130.

5. **Mabbott NA, Donaldson DS, Ohno H, Williams IR, Mahajan A.** 2013. Microfold (M) cells: important immunosurveillance posts in the intestinal epithelium. Mucosal Immunol **6:**666-677.

6. **Faherty CS, Redman JC, Rasko DA, Barry EM, Nataro JP.** 2012. *Shigella flexneri* effectors OspE1 and OspE2 mediate induced adherence to the colonic epithelium following bile salts exposure. Mol Microbiol **85:**107-121.

7. **Sistrunk JR, Nickerson KP, Chanin RB, Rasko DA, Faherty CS.** 2016. Survival of the Fittest: How Bacterial Pathogens Utilize Bile To Enhance Infection. Clin Microbiol Rev **29:**819-836.

8. **Nickerson KP, Chanin RB, Sistrunk JR, Rasko DA, Fink PJ, Barry EM, Nataro JP, Faherty CS.** 2017. Analysis of *Shigella flexneri* Resistance, Biofilm Formation, and Transcriptional Profile in Response to Bile Salts. Infect Immun **85**:e01067-16.

9. **Chanin RB, Nickerson KP, Llanos-Chea A, Sistrunk JR, Rasko DA, Kumar DKV, de la Parra J, Auclair JR, Ding J, Li K, Dogiparthi SK, Kusber BJD, Faherty CS.** 2019. *Shigella flexneri* Adherence Factor Expression in In Vivo-Like Conditions. mSphere **4**:e00751-19.

10. **Maurelli AT, Blackmon B, Curtiss R, 3rd.** 1984. Loss of pigmentation in *Shigella flexneri* 2a is correlated with loss of virulence and virulence-associated plasmid. Infect Immun **43:**397-401.

11. **Hromockyj AE, Maurelli AT.** 1989. Identification of *Shigella* invasion genes by isolation of temperature-regulated inv::lacZ operon fusions. Infect Immun **57:**2963-2970.

12. **Schroeder GN, Hilbi H.** 2008. Molecular pathogenesis of *Shigella* spp.: controlling host cell signaling, invasion, and death by type III secretion. Clin Microbiol Rev **21:**134-156.

13. **Tran QT, Gomez G, Khare S, Lawhon SD, Raffatellu M, Baumler AJ, Ajithdoss D, Dhavala S, Adams LG.** 2010. The *Salmonella enterica* serotype Typhi Vi capsular antigen is expressed after the bacterium enters the ileal mucosa. Infect Immun **78:**527-535.

14. **Miyoshi H, Stappenbeck TS.** 2013. *In vitro* expansion and genetic modification of gastrointestinal stem cells in spheroid culture. Nat Protoc **8:**2471-2482.
